# New insights on the Early Cretaceous (Hauterivian–Barremian) Urgonian lithostratigraphic units in the Jura Mountains (France and Switzerland): the Gorges de l’Orbe and the Rocher des Hirondelles formations

**DOI:** 10.1186/s00015-021-00395-5

**Published:** 2021-10-06

**Authors:** Antoine Pictet

**Affiliations:** Musée Cantonal de Géologie, 1015 Lausanne, Switzerland

**Keywords:** Rocher des Hirondelles Fm, Fort de l'Écluse Mb, Montcherand Mb, Bôle Mb, Rivière Mb, Urgonian carbonate platform

## Abstract

The Hauterivian–Barremian series of the Jura Mountains were measured over more than 60 sections along a 200 km long transect between Aix-les-Bains (Savoie Department, France) and Bienne (Bern Canton, Switzerland), which prompted the need for a revision and improvement of the current lithostratigraphic scheme for this stratigraphic interval. A new formation, the Rocher des Hirondelles Formation, is proposed in replacement of the unsuitable Vallorbe Formation, while the Gorges de l'Orbe Formation is formally described. The Gorges de l'Orbe Formation, equivalent to the well-known “Urgonien jaune” facies, consists of two members, namely Montcherand Member and Bôle Member. The Rocher des Hirondelles Formation, equivalent to the “Urgonien blanc” facies, consists of three members, i.e. Fort de l'Écluse Member, Rivière Member and Vallorbe Member. The marly Rivière and Bôle members appear to present time-equivalent lithostratigraphic units, recording a major sedimentological event affecting contemporarily both formations. This study proposes a new sedimentary model opening a new point of view on the long-living controversies about the age of the Urgonian series from the Jura Mountains. The data point to strong diachronic ages of lithostratigraphic units with a late Hauterivian to early Barremian occurrence of the “Urgonian blanc” facies in the Meridional Jura area versus a latest Barremian age in the Central Jura area, reflecting a general progradation of the Urgonian shallow-water carbonate platform from the present-day Meridional Jura area toward external deeper-water shelf environments of the present-day Central Jura area and Molasse basin.

## Introduction

The study of the Hauterivian and Barremian shallow-marine Urgonian series from the Jura Mountains has been very controversial from its inception, about 180 years ago, due to its highly complicated nature and the lack of benchmarks. The lithostratigraphic nomenclature did not ignore these controversies which opposed Marcou (1859) and de Tribolet (1859) as to the choice of the formation names and their type localities. This much criticized nomenclature quickly fell into oblivion to give way to more general and simplified nomenclatures, probably more adapted to the knowledge of the time and rather based on facies and stratigraphic positions (e.g. Desor & Gressly, 1859; Jaccard, 1869).

In the last decade, the Swiss Geological Survey has initiated the HARMOS project in order to supply spatial geological information nationwide. In the course of the project, the lithostratigraphic nomenclature was updated and homogenised for a target scale of 1:25,000 under the direction of the Swiss Committee of Stratigraphy (Strasky et al., 2016). At this occasion, Strasser et. al. (2016), who worked on the Lower Cretaceous series of the Jura Mountains, proposed the Gorges de l’Orbe Formation to replace facies terms such as ‘‘Urgonien jaune’’ (sensu Remane, 1989), “Urgonien inférieur” (Desor & Gressly, 1859) or “Russillien” (Jaccard, 1893), and the Vallorbe Formation to replace terms such as ‘‘Urgonien supérieur’’ or ‘‘Urgonien blanc’’ (Custer, 1928; Desor & Gressly, 1859).

Although reflecting the state of knowledge of the time, the new nomenclature of Strasser et. al. (2016) quickly appeared hardly applicable to the whole Jura mountain-range because of a stratigraphical dichotomy between the meridional and central Jura. Furthermore, this study did not provide a satisfactory answer to the strong age controversy surrounding the Urgonian series (Clavel et al., 2007, 2013, 2014; De Kaenel et al., 2020; Godet, 2006; Godet et al., 2010, 2011, 2012, 2013; Strasser et al., 2016) since the Gorges de l’Orbe and Vallorbe formations remained mostly dated with shallow-water benthic microfossils (Arnaud, 1981; Arnaud & Arnaud-Vanneau, 1991; Arnaud-Vanneau, 1980; Arnaud-Vanneau & Masse, 1989; Charollais et al., 1992, 2007, 2013a; Clavel et al., 2014). Furthermore, as all previous works, this proposition was based on two largely accepted historical axioms: (i) carbonate platform progradation follows a NE-SW axis based on the strong reduction and disappearance of lithological units in direction of the NE due to an hypothetic proximity of emerged lands (Adatte, 1988; Favre, 1843, 1867; de Loriol & Gilliéron 1869; Steinhauser, 1969; Steinhauser & Charollais, 1971); (ii) main lithostratigraphic units uniformly and contemporaneously deposited through the Jura realm (e.g. Strasser et al., 2016, p. 12).

**Fig. 1:**
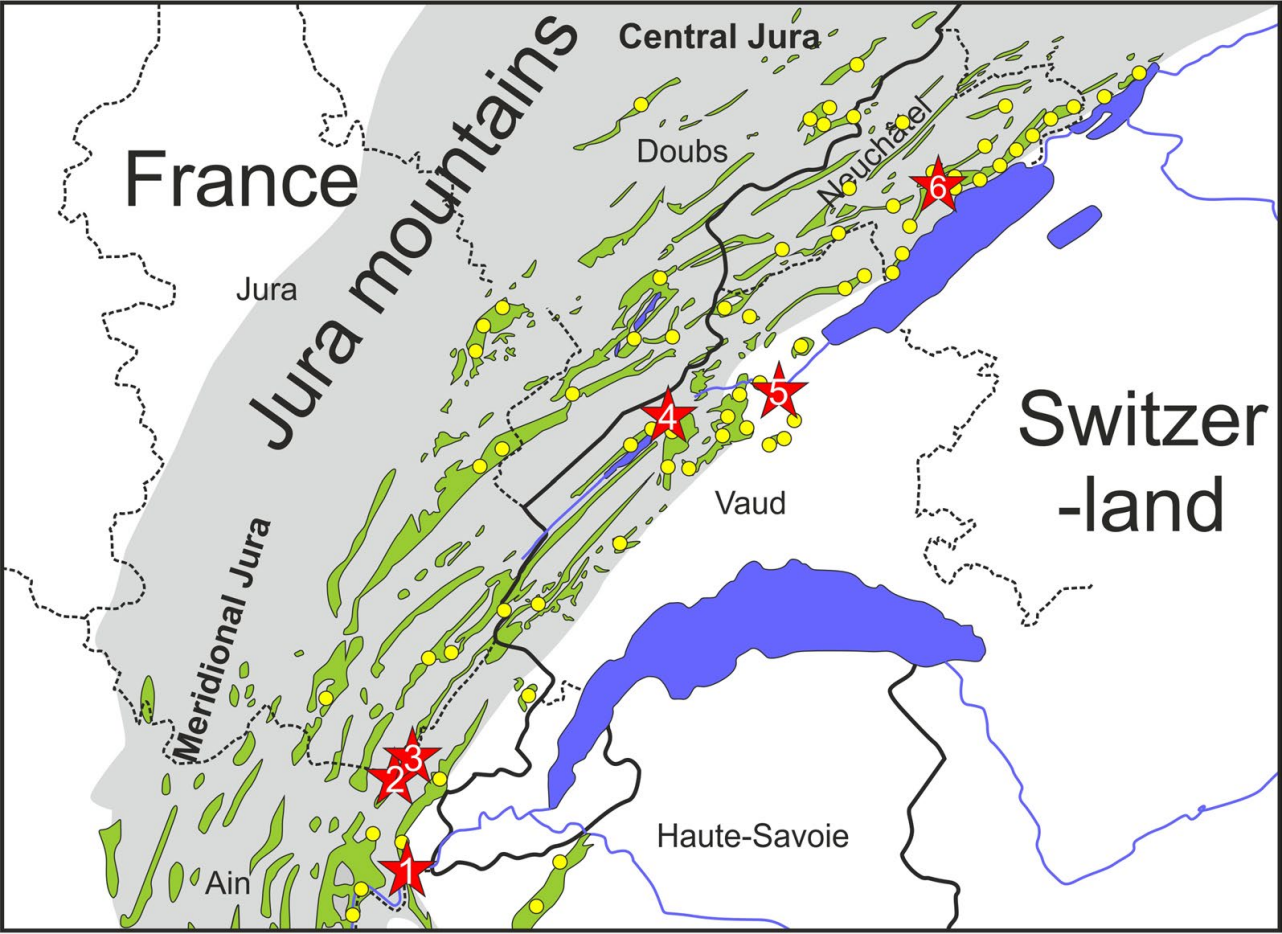
Geographical map of the Jura Mountains (eastern France and western Switzerland) with the location of the studied sections represented by yellow dots. Type-sections are located by the red stars; 1—Fort de l’Écluse; 2—Pont des Pierres; 3—Rocher des Hirondelles; 4—Vallorbe; 5—Montcherand (Gorges de l’Orbe); 6—Bôle. Modified after Strasser et. al. (2016)

The present study intends to reconcile the different interpretations by combining existing and new field data. The second starting point for this study was the hypothesis that shallow-water benthic microfossils such as foraminifers and green algae, largely responsible for the Urgonian age controversy, may be in fact facies dependent, and thus have been excluded from the present study. Between 2012 and 2019, more than 60 sections were investigated along a 200 km long transect between Aix-les-Bains (Savoie Department, France) and Bienne (Bern Canton, Switzerland) (Fig. 1). The new observations prompted for a revision of the current lithostratigraphic scheme of the Urgonian series.

## Materials and methods

The methods used in this work are in continuation of those described in Pictet et. al., (2016a).

Revised or new lithostratigraphic units of the Hauterivian to Barremian series are presently renamed in accordance with the rules of the International Stratigraphic Commission (ISC), in which each lithostratigraphic unit is defined by a geographic component, its lithological properties, and its stratigraphic relation (Hedberg, 1976; Remane et al., 2005). The present definition of new formations and members follows the state of the art, with historical context, type locality, thickness, boundaries, geographic distribution and biostratigraphy (using multiple markers).

**Fig. 2:**
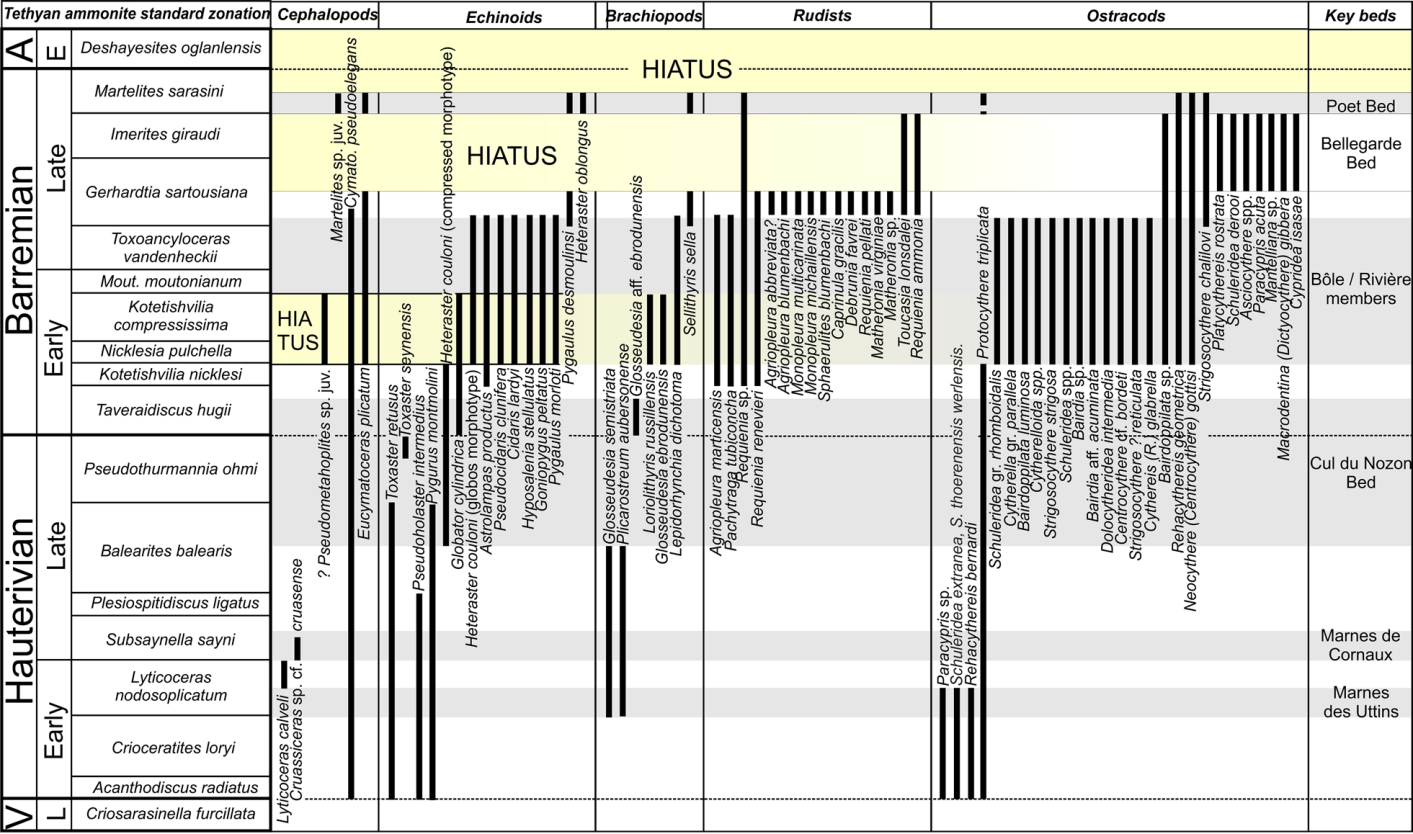
Ammonite zones according to Reboulet et al. (2018) and biostratigraphic distribution of main groups collected in the Hauterivian–Barremian series of Jura Mountains

The revision is based on traditional fieldwork (observations, sampling), microfacies analyses and interpretations, and biostratigraphy. Sections were logged and sampled at a variable sampling density (a few centimetres to several metres) depending on the lithological changes. A particular attention was given to the documentation of depositional geometries and discontinuity surfaces. Microfacies analyses, sequence stratigraphy, sedimentology and ammonite biostratigraphy were applied to each section. The microfacies has been determined on hundreds of thin sections to describe the environmental changes in order to infer the eustatic variations. The collected and/or studied macro- and microfauna, which is characterized by cephalopods (ammonites, nautiloids, belemnites), gastropods, bivalves, echinoids, serpulids, brachiopods, sponges, corals, ostracods, benthic foraminifers, dinocysts and nannofossils were used to date and to infer environmental conditions. Ammonite biostratigraphy has been based on the zonal scheme of the Tethyan region proposed by the IUGS Lower Cretaceous ‘‘Kilian Group’’ during the 6th International Meeting (Fig. 2, Reboulet et al., 2018). Ammonite biozones reported on the figures read as follows: biozones verified with ammonites are indicated in black characters on grey background; biozone verified with ammonites on other sections reported by correlations are indicated in grey characters; unverified biozones suggested on sequence stratigraphic interpretations and by other fossils of biostratigraphic value are indicated in grey characters between brackets. Additional fossils stored in the museums of Geneva (collections Générale, Jaccard, de Loriol, de Mortillet, Mouty, Pictet F. J., Rochat, Steinhauser), Lausanne (collections Geol. Reg., Pal. Syst.), Neuchâtel (collections Baumberger, Junod, stratigraphique générale), Basel, Lyon (collections Busnardo, Charollais, Clavel and Charollais, Clavel), and Fribourg (collection Schardt) were also documented. All the collected material (rock samples, thin sections, fossils) is deposited at the Museum of Lausanne, collection A. Pictet.

Major discontinuity surfaces were identified and correlated as precisely as possible. They are indicated on the logs and figures and numbered with D.Ha for Hauterivian discontinuities and D.Ba for Barremian discontinuities. These discontinuity surfaces were correlated to the sequence stratigraphic scale from Arnaud (2005), based on the Barremian stratotype of Angles (SE France). However, an additional key surface is recognized at the boundary between the *T. vandenheckii* and the *G. sartousiana* ammonite zones, also identified in Tendil et. al. (2018) and Frau et. al. (2021). This surface is currently interpreted as a sequence boundary, labelled Sb Ba3′.

The present research was submitted to several reviewers, Dr. Alain Morard, Dr. André Strasser, Dr. Hanspeter Funk, Dr. Reto Burkhalter, Dr. Danielle Decrouez, and presented on March 26th to the Swiss Committee of Stratigraphy for approval. The interpretations and the proposed lithostratigraphic nomenclature were fully supported by the committee.

Some of the field work was realized in the company of Dr. Pierre-Olivier Mojon between 2014 and 2019. Following a difference of opinion on the interpretation of the data, each party will carry out its own study in his field of competence. Numerous marly levels were sampled for their micropaleontological content, which were washed and analysed by Dr. P.-O. Mojon. A detailed and up to date study of the ostracods of the Urgonian facies will be published by Dr. P.-O. Mojon.

## Gorges de l'Orbe Formation (GDO Fm)

### Definition

The Gorges de l’Orbe Formation was proposed by Strasser et. al. (2016) to replace facies-based terms such as ‘‘Urgonien jaune’’ (sensu Remane, 1989), “Urgonien inférieur” (Desor & Gressly, 1859) or “Russillien” (Jaccard, 1893).

**Fig. 3:**
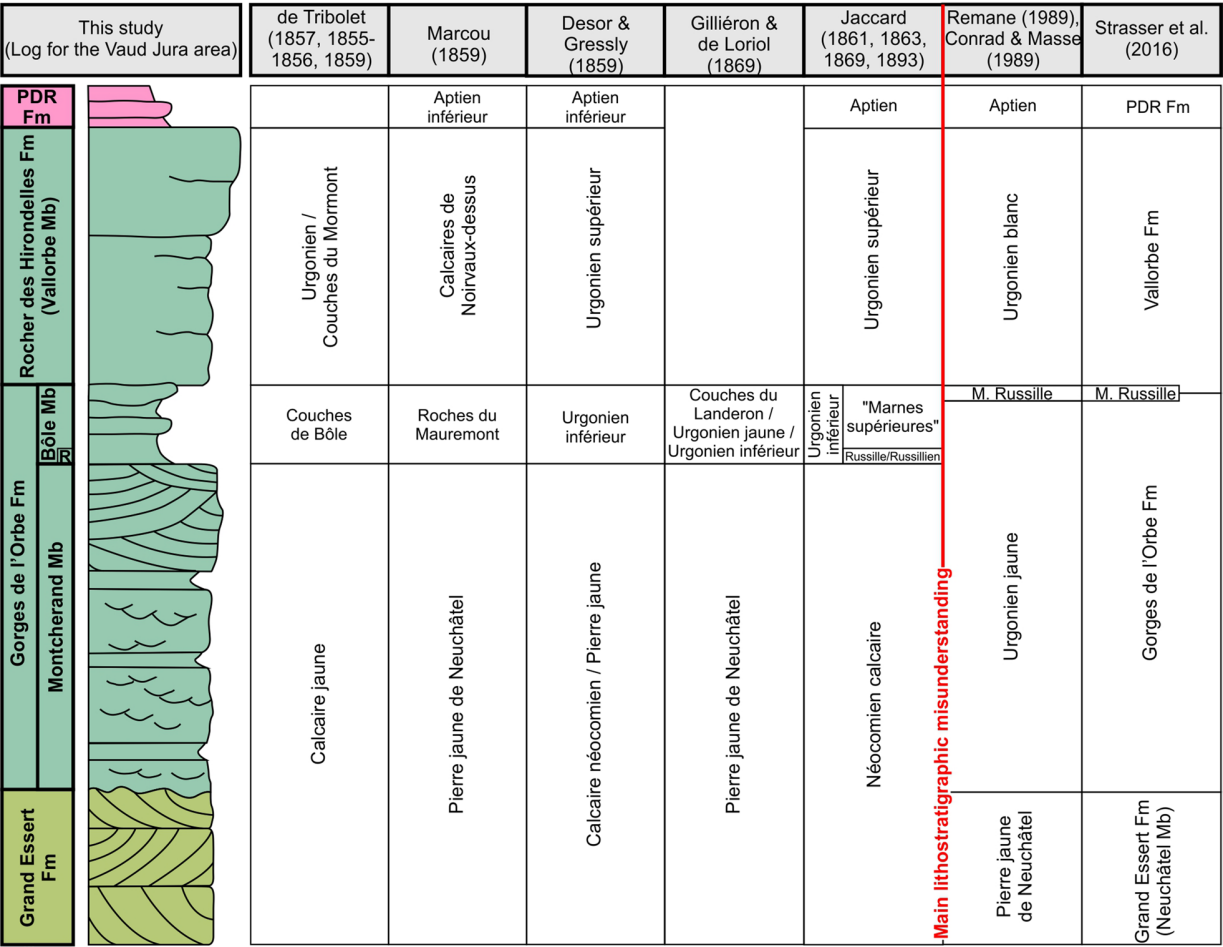
Nomenclatural historic of main terms used to describe Upper Hauterivian to Lower Aptian series in the Central Jura. Lithological log modified after Godet et. al. (2010)

The first mention of the “Calcaire urgonien jaune” by Campiche and de Tribolet in Campiche & Pictet (1858) cannot be assigned to a precise stratigraphic interval (Fig. 3). Later, de Loriol & Gilliéron (1869) clearly used the ‘‘Urgonien jaune’’ term as an equivalent of the “Urgonien inférieur” of Desor and Gressly (1859) (Fig. 3). Jaccard (1863, 1869) introduced the “Marne(s) de la Russille” level in reference to a rich fauna delimiting the boundary between the “Urgonien inférieur” and the “Néocomien calcaire” (Fig. 3). The important dissertation ‘‘Révision de l'étage hauterivien (région-type et environs, Jura franco-suisse)’’ by Remane (1989) attempted to synthesize the knowledge of the time about the Hauterivian–Barremian series of the Jura Mountains. Unfortunately, the authors interpret the historical literature in an erroneous way, especially about the lithostratigraphic position of Jaccards’ “Marne(s) de la Russille”. This led Remane (1989) to attribute the “Urgonien jaune” to a limestone succession with numerous marly intercalations, situated stratigraphically below the key level of Jaccard, a stratigraphic interval which was until that time part of the “Pierre jaune de Neuchâtel” facies or “Néocomien calcaire” (Desor, 1873; Desor & Gressly, 1859; Jaccard, 1869; Schardt, 1891; Schardt & Dubois, 1902; Fig. 3). Thus, a new “Urgonien jaune” unit was born with the ‘‘Révision de l'étage hauterivien’’ (Remane, 1989), leading to a major ambiguity. At the same time, and for the same reasons, Conrad and Masse (1989, p. 311) noted the presence at the Russille type-locality of a topmost marly fossiliferous level, which they thought to correspond to the historical level of the Marne de La Russille (see also Clavel et al., 1994, p. 35; Fig. 3). As a result, Strasser et. al. (2016) enclosed the Marnes de la Russille sensu Conrad and Masse (1989) into the base of the “Vallorbe Formation” (Fig. 3).

The GDO Fm of Strasser et. al. (2016) thus includes the “Urgonien jaune” (sensu Remane, 1989, i.e. below the Marne de la Russille sensu Jaccard, 1869) and most of the “Urgonien inférieur” of Desor and Gressly (1859). In order to solve this lithostratigraphic mishmash, we presently emend the Gorges de l’Orbe Formation to incorporate all levels formerly assigned to one or the other acceptation of the Marnes de la Russille or “Russilien” (sensu Jaccard, 1893), hence encompassing the “Urgonien jaune” (sensu Remane, 1989) in its lower part and the “Urgonien inférieur” (sensu Desor & Gressly, 1859) in its upper part, and turn back to the initial definition of the Marne(s) de la Russille by Jaccard (Fig. 3). The base of the overlying “Vallorbe Formation” is emended consequently.

#### Type locality

**Fig. 4:**
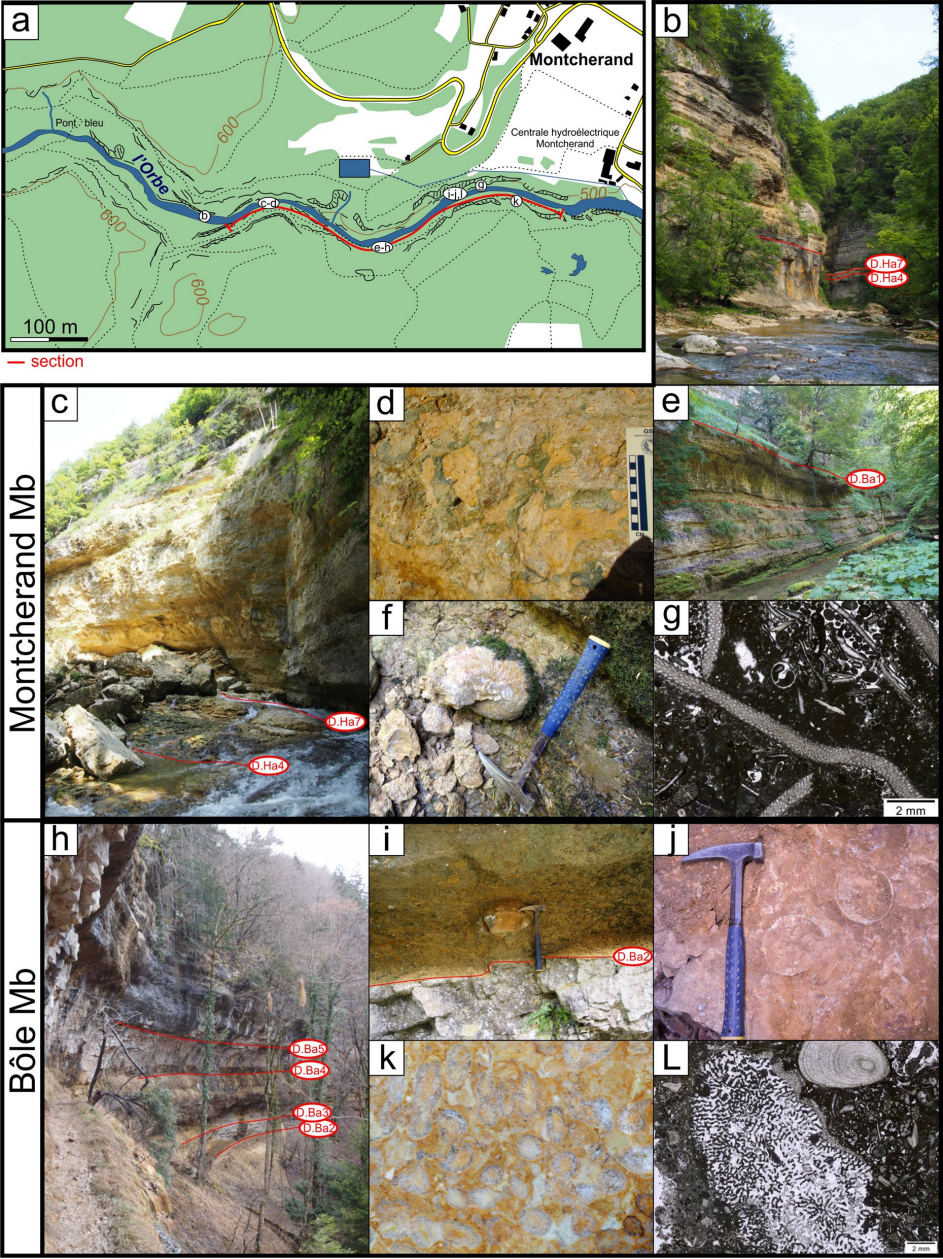
**a** Location map of the type-section of the Gorges de l’Orbe Formation at Montcherand (Vaud, Switzerland). Moncherand Member: **b** transition between the Grand Essert Formation (Neuchâtel Member) and the Gorges de l’Orbe Formation, **c** lowermost series of the Gorges de l’Orbe Formation with the Pont des Pierres and La Vaux beds; **d** focus on the glauconitic hardground at the top of the Pont des Pierres Bed; **e** Focus on the La Vaux Bed; **f** *Cymatoceras pseudoelegans* (d’Orbigny 1840) from the La Vaux Bed; **g** Bryozoan-rich muddy facies of the La Vaux Bed. Bôle Member: **h** view of the Bôle Member; **i** Discontinuity surface D.Ba2 at the transition between the Montcherand Member and the Russille Bed; **j** Oysters encrusting the discontinuity surface D.Ba2; **k** polished slab with corals from the TST of the uppermost marly intercalation of the Bôle Member; **l** muddy facies of the Bôle Member with corals and stromatoporoids

The type locality is well exposed on a 1.3 km-long natural outcrop extending on both sides of the Orbe river valley, between the Montcherand hydro-electrical station and the “Pont bleu” (Fig. 4; Coordinates 2529′389/1175′470 to 2528′412/1175′527 system CH1903/LV03). Detailed description of this locality was given by Conrad & Masse (1989) and Blanc-Alétru (1995).

#### Thickness

The GDO Fm presents its maximum thickness at the type locality with approximatively 56 m (Strasser et al., 2016, Fig. 5). Nevertheless, strong lateral thickness and facies changes are observed on only few kilometres following a succession of depocenters and topographic highs delimited by synsedimentary faults.

**Fig. 5:**
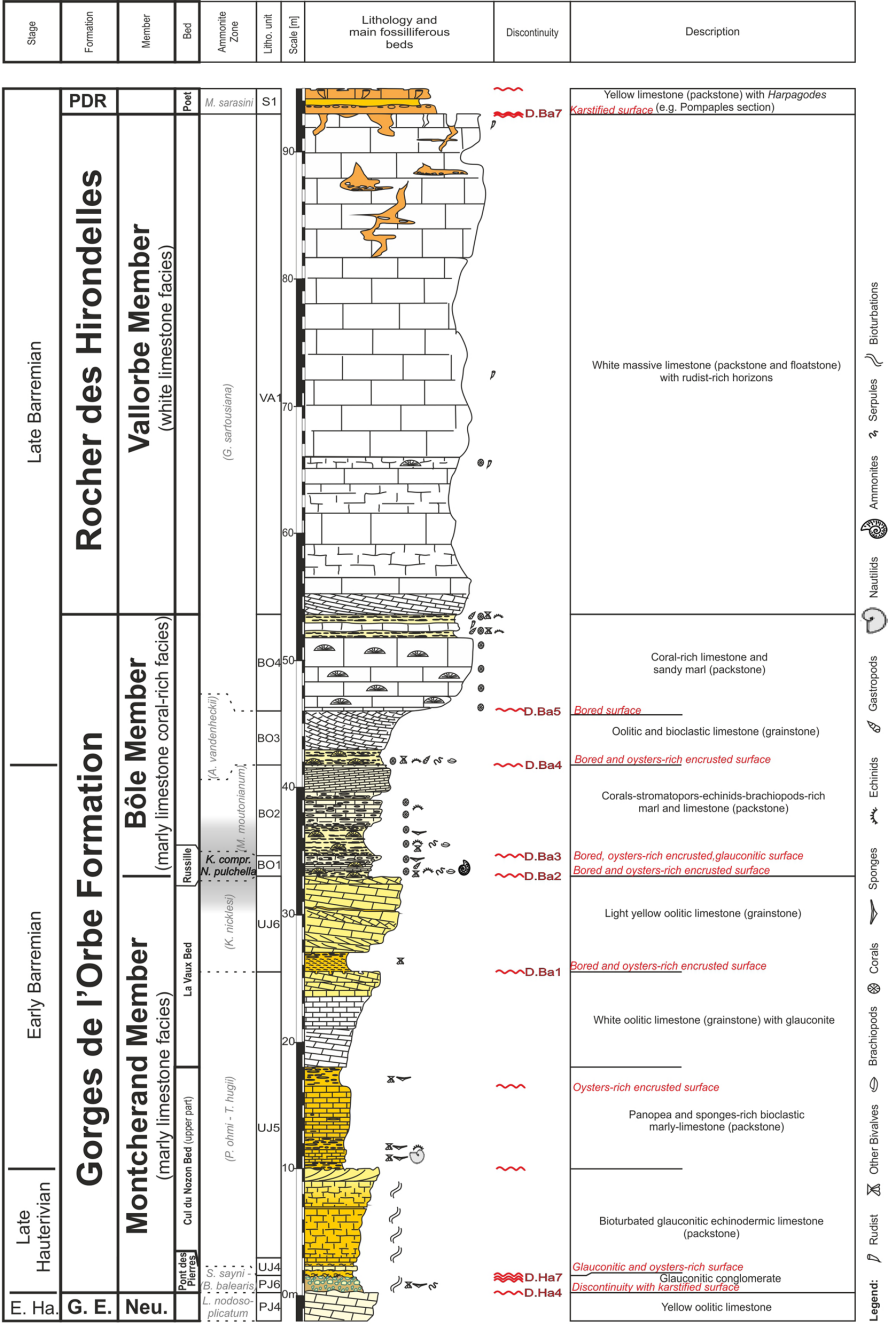
The type-section of the Gorges de l’Orbe Formation at Montcherand with age, litho- and biostratigraphy on the left side, and the main fossiliferous beds, discontinuity surfaces and description of the log on the right side

#### Definition and boundaries

The GDO Fm overlies the Grand Essert Formation (Pierre jaune de Neuchâtel facies *sensu* Remane, 1989, Neuchâtel Member in Strasser et al., 2016) by the intermediary of a discontinuity surface D.Ha3 in the Meridional Jura, D.Ha4 in the western Central Jura, D.Ha5 or D.Ha6 in the eastern Central Jura and so one passing in great part to the Pierre jaune de Neuchâtel facies. This diachronous boundary may be difficult to distinguish in the field since both formations display similar facies with various amounts of ooids (Blanc-Alétru, 1995; Remane, 1989; Rumley, 1993; Strasser et al., 2016). Nevertheless, both formations, which are separated by clear hardgrounds, can be distinguished more easily with the following criteria. TheNeuchâtel Member is exclusively characterized by yellow, well bedded, homogenous, massive limestones (Marcou, 1859; Jaccard, 1869). The GDO Fm characterizes on the field by softer cliffs with numerous depressions. The lower boundary itself is marked by the presence of: (i) glauconite-rich levels (packstone); (ii) marly or more marly-limestone levels; (iii) abundant sponge spicules and limestone nodules (mostly sponges) as well as abundant bivalves related to the genus *Panopea* Menard 1807. The GDO Fm is characterised by outer platform, nodular, yellow, marly-limestone facies and by its fauna dominated by sponges and muddy bivalves.

#### Geographic distribution and lateral equivalents

The GDO Fm is restricted to the Jura Domain. It passes westward to the Fort de l'Écluse Member (see Sect. 4.1). Eastward, like in the Morteau and Combes-Le Landeron areas, the GDO Fm shows a peculiar aspect that Jaccard (1870, p. 27) called the “Calcaire jaune de Morteau”. This atypical facies alternates at its base with oolitic limestone digitations of the Grand Essert Formation and continues above the Bôle Member with a yellow limestone without rudist-shells which no longer shares any character with the “Urgonien blanc facies” (Jaccard, 1870, pp. 27–28). More distantly, the GDO Fm is a time-equivalent to the Helvetic Tierwis Formation (Föllmi et al., 2007), and southward to the Subalpine “Formation des Calcaires siliceux” (Pairis et al., 1993) *pro parte* and southward to the Urgonian Formation (Arnaud-Vanneau & Arnaud 1990) *pro parte*.

#### Biostratigraphic data and age

Biostratigraphic ages, detailed in the next chapters, are mostly referring to published data on ostracods, nannoflora, dinocysts, echinoids, rudist shells, and rare cephalopods findings. Biostratigraphic fossils indicate that the GDO Fm covers series from: (i) the late early Hauterivian (*L. nodosoplicatum* Zone) to the latest Hauterivian (*B. mortillei* Zone) in the Meridional Jura; (ii) the late Hauterivian (*S. sayni* Zone) to the middle late Barremian (lower *G. sartousiana* Zone) in the western eastern Central Jura; (iii) the late early Barremian (*N. pulchella* Zone) to the latest Barremian (upper *G. sartousiana* Zone) in the eastern Central Jura.

### Montcherand Member

This new member corresponds to the “Urgonien jaune” of Remane (1989), a marl and limestone series petrographically and paleontologically close to the “Pierre jaune de Neuchâtel” facies (Fig. 3). Although we presently give a definition of the “Urgonien jaune” facies, the lower boundary is sometimes difficult to place, or even arbitrary, especially in the Neuchâtel region where the two facies intertwine (Blanc-Alétru, 1995; Remane, 1989; Rumley, 1993; Strasser et al., 2016).

#### Type locality

As for the GDO Fm, the Montcherand Member is well exposed upstream the Montcherand hydro-electrical station (Fig. 4b, c–e; Coordinates 2529′229/1175′500 to 2528′412/1175′527 system CH1903/LV03) and was described in detailed by Conrad & Masse (1989) and Blanc-Alétru (1995).

#### Thickness

The Montcherand Member presents its maximum thickness at the type locality with approximatively 33 m (Strasser et al., 2016; Fig. 5). As for the GDO Fm, strong lateral thickness and facies changes are observed in only few kilometres following a succession of depocenters and topographic highs, the member even being locally absent (e.g. Northern side of Mt Chamblon, Bôle, Le Landeron) or replaced by the “Pierre jaune de Neuchâtel” facies.

#### Definition and boundaries

The Montcherand Member is commonly bioturbated, channelized (e.g. Cul du Nozon section), and presents numerous truncated surfaces with hardgrounds. The texture is generally bioclastic, partly oolitic with few detrital quartzes and glauconite components. The biota, autochthonous or sub-autochthonous, is typical of shallow-water to deeper open-marine habitats with cephalopods (Fig. 4f), bivalves, echinids, crinoids, bryozoans (Fig. 4g) and sponges. Westward outcrops, represented by shallower environments, deliver rare scleractinian corals, large benthic foraminifera including rare orbitolinids, and dasycladalean algae.

#### Subdivisions, geographic distribution and lateral variations

The Montcherand Member usually consists of 3 successive subunits which are from bottom to top: (i) the Pont des Pierres Bed; (ii) the Cul du Nozon Bed, (iii) the La Vaux Bed.

**Fig. 6:**
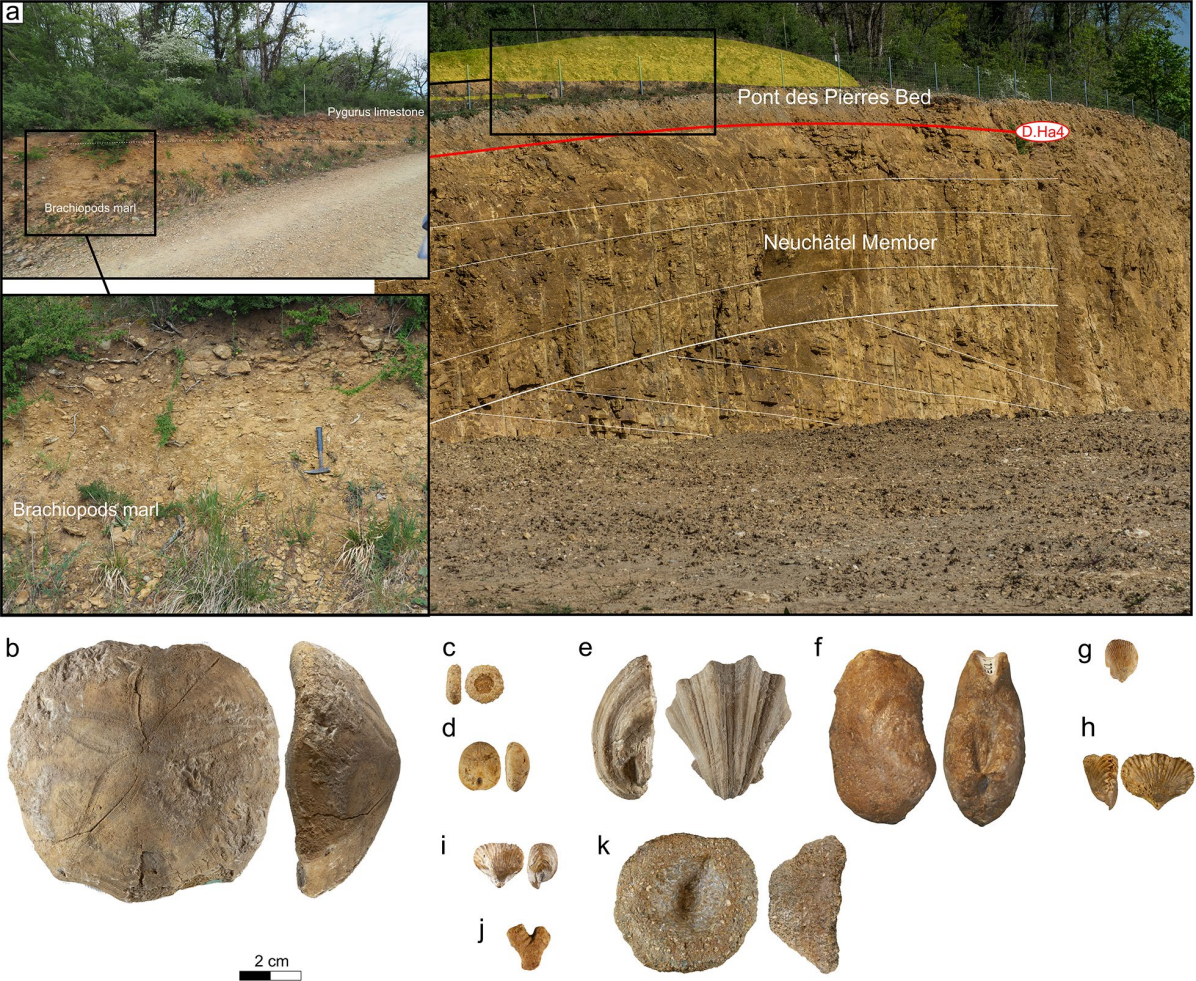
**a** The strongly glauconitic Pont des Pierres Bed at the top of the Eclépens quarry (Vaud canton, Switzerland) topping the cross-stratified oolithic Pierre jaune de Neuchâtel (PJN). Coordinates 2530′814/1167′761 system CH1903/LV03. **b** *Pygurus montmollini* Agassiz 1839, Tylonne, Vaud, Switzerland, MGL.107279 coll. Paris. **c** *Pseudodiadema* sp., Eclépens, Vaud, Switzerland, MGL.107325 coll. A. Pictet. **d** *Nucleolites scheuchzeri* (Desor in de Loriol 1873), Eclépens, Vaud, Switzerland, MGL. 107326 coll. A. Pictet. **e** *Neithea atava* (Roemer 1839), Tylonne, Vaud, Switzerland, MGL.21816 coll. Paris. **f** *Panopea neocomiensis* (Leymerie 1842), Eclépens, Vaud, Switzerland, MGL.107281 coll. A. Pictet. **g** *Limatula tombeckiana* (d’Orbigny 1845), Eclépens, Vaud, Switzerland, MGL.107327 coll. A. Pictet. **h** *Plicarostrum aubersonense* Burri, 1956, Rochefort, Neuchâtel, Switzerland, MGL.107280 coll. A. Pictet. **i** *Lamellaerhynchia hauteriviensis* Burri 1953, Eclépens, Vaud, Switzerland, MGL. 107328 coll. A. Pictet. **j** Bryozoan, Eclépens, Vaud, Switzerland, MGL. 107329, coll. A. Pictet. **k** Eponge, Eclépens, Vaud, Switzerland, MGL.107282 coll. A. Pictet

The Pont des Pierres Bed is a basal glauconitic marl-limestone complex (wacke- to packstone; Fig. 4d; = Bancs à panopées, Revil, 1911). This unit is mostly present in the Meridional Jura where it can reach up to 30 m thickness and build the main part of the GDO Fm with an alternation of slightly glauconitic units characterized by marls grading upward to a limestone unit presenting planar, cross-bedded and in trough stratifications (e. g. Mehno 2013). At the Grand Essert, the lowermost layers delivered to Strasser et. al. (2018) various ostracods as *Cythereis*? sp., *Paracypris* sp., *Protocythere triplicate* (Roemer 1841), *Schuleridea extranea* Grosdidier 1964, and *S. thoerenensis werlensis* Gründe 1966. Toward the Central Jura, this unit progressively becomes thinner and occurs sporadically along the SW–NE axis, mostly restricted in lowered compartments of tilted blocks. This deposit is strongly condensed and extremely glauconitic and delivers taxa as *Pseudoholaster intermedius* (Münster in Goldfuss 1826), *Pygurus montmollini* (Agassiz 1836) (Fig. 6b), *Pseudodiadema* sp. (Fig. 6c), *Nucleolites scheuchzeri* (Desor in de Loriol 1873) (Fig. 6d), *Neithea atava* (Roemer 1839) (Fig. 6e), *Panopea neocomiensis* d’Orbigny (Fig. 6f), *Limatula tombeckiana* (d’Orbigny 1845) (Fig. 6g), *Plicarostrum aubersonense* Burri 1956 (Fig. 6h), *Lamellaerhynchia hauteriviensis* Burri 1953 (Fig. 6i), bryozoans (Fig. 6j), and sponges (Fig. 6k). A good outcrop of the Pont des Pierres Bed is situated on top of the northern side of the Eclépens quarry, where very glauconitic marls and limestones crop out (Fig. 6a), separated from the rest of the quarry by a large fault. Further eastward, the Pont des Pierres Bed laterally passes to the Grand Essert Formation with cross-bedded oolitic limestones intercalated with sponge-rich marls (e.g. sections around Neuchâtel). This unit ends by a well-marked and perforated hardground (D. Ha6), strongly enriched in phosphate.

**Fig. 7:**
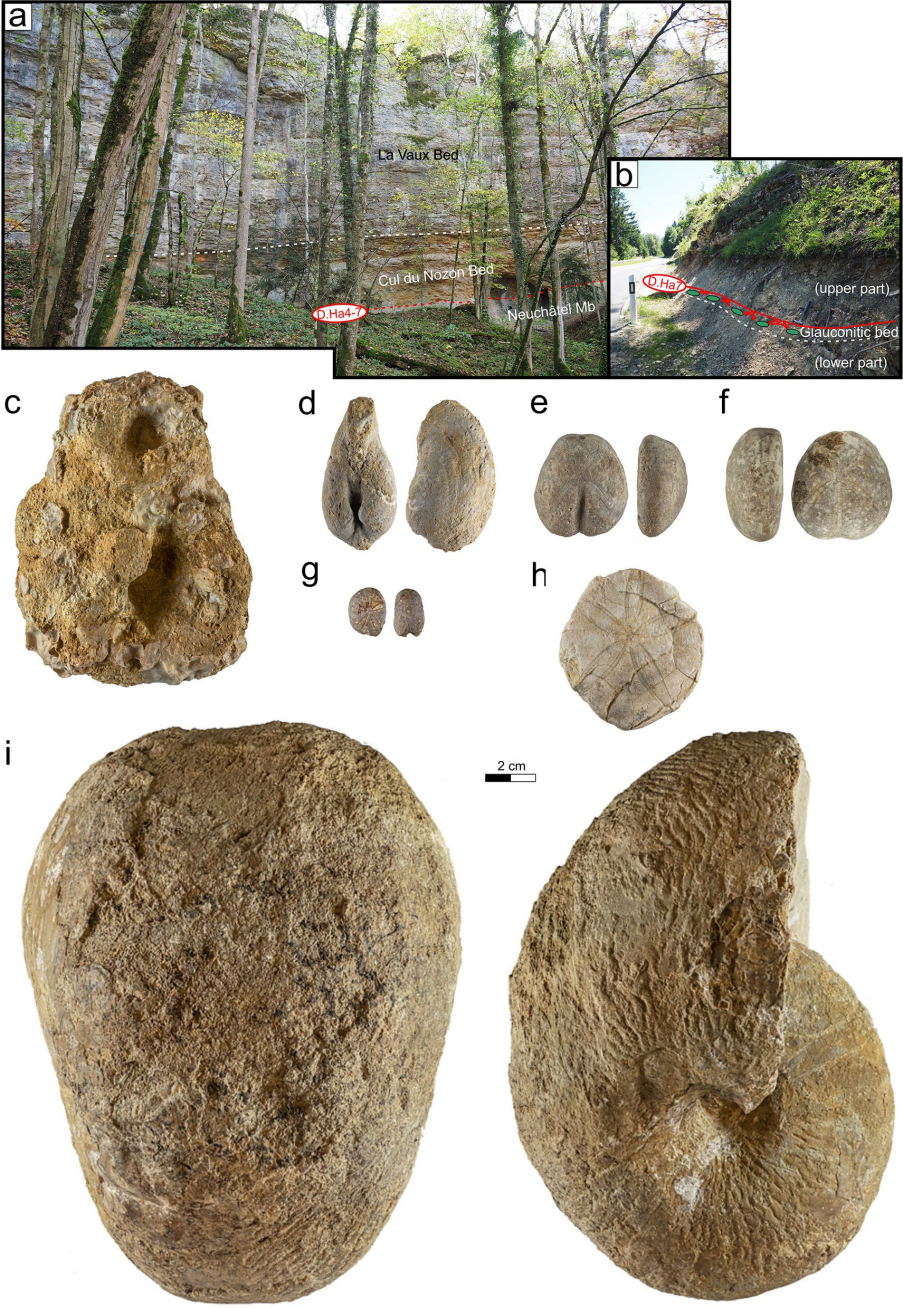
**a** General view of the Vallée d’Engens outcrop in the Gorges du Nozon (Coordinates 2526′553/1168′782 system CH1903/LV03) showing the Cul du Nozon Bed framed below by the Grand Essert Formation and above by the La Vaux Bed. **b** Focus on the Cul du Nozon Bed at their type locality (coordinates 2518′963/1170′674 system CH1903/LV03) showing a glauconitic firmground separating the uppermost Hauterivian series from the lowermost Barremian series. **c** Sponge, Bois de Fives, Gorges de l’Orbe, Vaud, Switzerland, MGL.107283 coll. A. Pictet. **d **Panopea *neocomiensis *(Leymerie 1842), Déchèterie du Bugnon, Vallorbe, Vaud, Switzerland, MGL.108836 coll. A. Pictet. **e** *Toxaster retusus* (Lamarck 1816), La Chambotte, Haute-Savoie, France, MGL.107284 coll. A. Pictet. **f** *Heteraster couloni* Agassiz 1839, compressed morphotype, La Chambotte, Haute-Savoie, France, MGL.107285 coll. A. Pictet. **g** *Globator cylindrica* (Gras 1848), Cul du Nozon, Vaud, Switzerland, MGL.107286 coll. A. Pictet. **h** *Astrolampas productus* (Agassiz 1836), Le Bugnon, Vallorbe, Vaud, Switzerland, MGL.107287 coll. A. Pictet. **i** *Cymatoceras pseudoelegans* (d’Orbigny 1840), Montcherand, Gorges de l’Orbe, Vaud, Switzerland, MGL.101513 coll. A. Pictet

The above Cul du Nozon Bed is represented by a *Panopea*-rich, blue-gray marl-dominated unit (packstone, Fig. 7a). In the Meridional Jura, this unit was confused with the Marne de la Russille as it is directly overlain by the Urgonien blanc facies (Blondel, 1990; Conrad, 1968, 1969). It grades upward to the “oolithe caviar”, a particularly remarkable oolithic grainstone at the base of the Fort de l’Écluse Mb, and in the Central Jura to a nodular marly-limestone. “Nodules” or “pebbles”, as they were interpreted by previous authors (e.g. Blanc-Alétru, 1995), are in fact mostly composed of autochtonous reworked sponges (Fig. 7c) and strongly bioturbated pre-indurated sediments with numerous *Panopea neocomiensis* d’Orbigny (Fig. 7d). In the Chambotte section, the Cul du Nozon Bed delivered echinoids such as *Toxaster retusus* Lamarck 1816 (Fig. 7e) and a primitive compressed morphotype of *Heteraster couloni* (Agassiz 1839) (Fig. 7f). A *Toxaster seynensis* (Lambert 1920) was collected in the Valserine valley. In the central Jura, the Cul du Nozon Bed is divided in its center by a glauconite-rich firmground (Fig. 7b). The part below the glauconitic firmground, which is present only in few sections, did not deliver fossils, whereas the upper part delivered brachiopod s, few gastropods, the echinoid *Globator cylindrica* (Gras 1848) (Fig. 7g) and nautilids as *Cymatoceras pseudoelegans* (d’Orbigny 1840) (Fig. 7i).

The La Vaux Bed progressively derives from the Cul du Nozon Bed and is characterized by a yellow to white, planar to cross-bedded, massive, oolitic limestone unit (pack- to grainstone, Figs. 4b, 7a), which grades south-westward to the rudist-rich limestones of the Fort de l'Écluse Member described below. Few coral reefs were observed in this unit of the Vaud Jura area, at the transition between the Fort de l'Écluse and the Moncherand members. The La Vaux Bed ends with a truncated, karstified surface (D.Ba2). The fauna, still rare, is represented by few echinoids such as *Astrolampas productus* (Agassiz 1836) (Fig. 7h), *Heteraster couloni* (Agassiz 1839), and nautilids as *Cymatoceras* sp.

Another facies with 3 m of yellow massive and glauconite-rich limestones was observed in the Eclépens quarry. Two ammonites were found by foremen, a *Cruasiceras* cf. *cruasense* (Torcapel 1884) and *Lyticoceras claveli* Busnardo & Thieuloy 1983, just on top of the Neuchâtel Member. These condensed sediments, possibly representing a very condensed equivalent of the Montcherand Mb, are topped by the dark marls of the Bôle Member.

#### Biostratigraphic data and age

Still accessible, the quarries of Bôle have recently been transformed into a residen The lowermost marly intercalations of the Pont des Pierres Bed of the Grand Essert section, Meridional Jura, delivered to Strasser et. al. (2018) ostracods and dinokysts associations which indicate a latest early Hauterivian age (*L. nodosoplicatum* ammonite Zone), an age sustained by the presence of characteristic ammonites in neighbouring sections. The authors reported these lowermost marly layers to the Marne des Uttins. Still at the Grand Essert section, an ammonite was collected in the middle of the member by Mouty (1966), which is attributed to the genus *Cruasiceras* Busnardo 1970, dating the lowermost upper Hauterivian *S. sayni* Zone (see also Clavel et al., 2007; Strasser et al., 2018). This ammonite was collected in association with the echinoid *Toxaster retusus* Lamarck 1816 indicating an Hauterivian age. Other fossil groups present in the Cul du Nozon Bed such as echinoids with *Pygurus montmollini* (Agassiz 1836), *Pseudoholaster intermedius* (Münster in Goldfuss 1826) and brachiopods with *Plicarostreum aubersonense* Burri, 1956 also clearly indicate an Hauterivian age (Burri, 1956; Clavel, 1989; Strasser et al., 2018).

The Cul du Nozon Bed of the La Chambotte section shows the combined presence of the echinid *Toxaster retusus* Lamarck 1816 and *Heteraster couloni* (Agassiz 1839), indicating a late Hauterivian age not younger than the *B. balearis* ammonite Zone (Charollais et al., 2009; Clavel et al., 2014). In the Valserine valley, the presence of a primitive morphotype of *Toxaster seynensis* (Lambert 1920) in the basal part of the Cul du Nozon Bed indicates a late Hauterivian age not older than the *P. ohmi* ammonite Zone or the earliest Barremian (Clavel in Charollais et al., 2009). Conrad (1969, p. 7) reported this marly interval to the base of the Barremian or straddling the Hauterivien-Barremian boundary. The dark marls from the base of the Cul du Nozon Bed in the Morand drilling core delivered to Jan du Chêne et. al. (2016) a dinokysts association of late Hauterivian age. In the eastern Central Jura, the Cul du Nozon Bed delivered the echinoid *Globator cylindrica* (Gras 1848), a typical Barremian species. The Cul du Nozon Bed is therefore a diachronous level straddling the Hauterivian–Barremian boundary (Conrad, 1968, 1969) with a latest Hauterivian age in the Meridional Jura, and an earliest Barremian age in the Central Jura.

The La Vaux Bed did not yield determinable cephalopods. The echinoid *Astrolampas productus* (Agassiz 1836), present in the median marly intercalation is a typical Barremian taxon. The very local yellow massive and glauconite-rich limestones of the Eclépens quarry delivered at their base a nannoflora interpreted by De Kaenel et. al. (2020) from the *T. hugii* Zone, which led the authors to suggest that both hauterivian ammonites were reworked in this unit during the earliest Barremian. The internal mold of a body chamber of *Lyticoceras claveli* Busnardo & Thieuloy 1983 is made by an oolithic grainstone typical of the underlying Neuchâtel Member. This ammonite dates from the early Hauterivian *L. nodosoplicatum* Zone. The second ammonite, a negative imprint of *Cruasiceras* cf. *cruasense* (Torcapel 1884), is made by the yellow massive and glauconite-rich limestone, and dates from the early Late Hauterivian *S. sayni* Zone.

### Bôle Member

The “Couches de Bôle” were first used by de Tribolet (Baumberger, 1898; de Tribolet 1856, 1856–1857, 1857, 1859; de Tribolet in Jaccard, 1869, p.141; Desor & Gressly, 1859; Fig. 3) for the fossiliferous marly-limestones cropping out in the quarries of Bôle near Neuchâtel, a famous fossil locality (Renevier, 1853; Schardt & Dubois, 1902). In the same period, Marcou (1859) called these levels “Roches du Mauremont (= Mormont ! NDLR; Fig. 3), a denomination rejected by de Tribolet (1859) because inadequate. In their study of the Neuchâtel Jura, Desor and Gressly (1859) described these coral- and echinid-rich yellow marly limestones as “Urgonien inférieur” (Fig. 3). Some years later, de Loriol & Gilliéron (1869) published the palaeontological and stratigraphical monography of the “Urgonien inférieur or Urgonien jaune” stage of Le Landeron (NE) which they formally named “Couches du Landeron”. Their type section has disappeared due to urbanization. Jaccard (1861, p. 77) described a 10-cm-thick yellow Panopea-rich limestone just below the “Urgonien inférieur”, characterized by the large brachiopod “*Terebratula semistriata*” (revised as *Terebratula ebrodunensis* in Jaccard, 1869). Jaccard (1863, p. 10) finally integrated this *Terebratula ebrudunensis*-rich marly bed to the lower part of the “Urgonien inférieur” marking the boundary with the “Neocomien calcaire” as it was defined at the time, and called this layer “Marne(s) de la Russille” in reference to the rich fauna he collected on the type-section. The “Marnes de la Russille” definition was later distorted by Schardt and Dubois (1902) in an expanded meaning for the whole “Urgonien inférieur” and for the Fort de l’Écluse Member (Sect. 4.1) which he corelated together. Conrad and Masse (1989) also expanded its meaning for the whole “Urgonien inférieur”, and like Blondel (1990), called “Marnes de la Russilles” all yellow marly limestones situated below the “Urgonien blanc” regardless of the region and the age. This last localisation just below the “Urgonien blanc” lead in turn Clavel et. al., (1994) to misinterpret Jaccard’s description of the type locality and to move the Marne de la Russille Bed on top of the Bôle Member.

#### Type locality

**Fig. 8:**
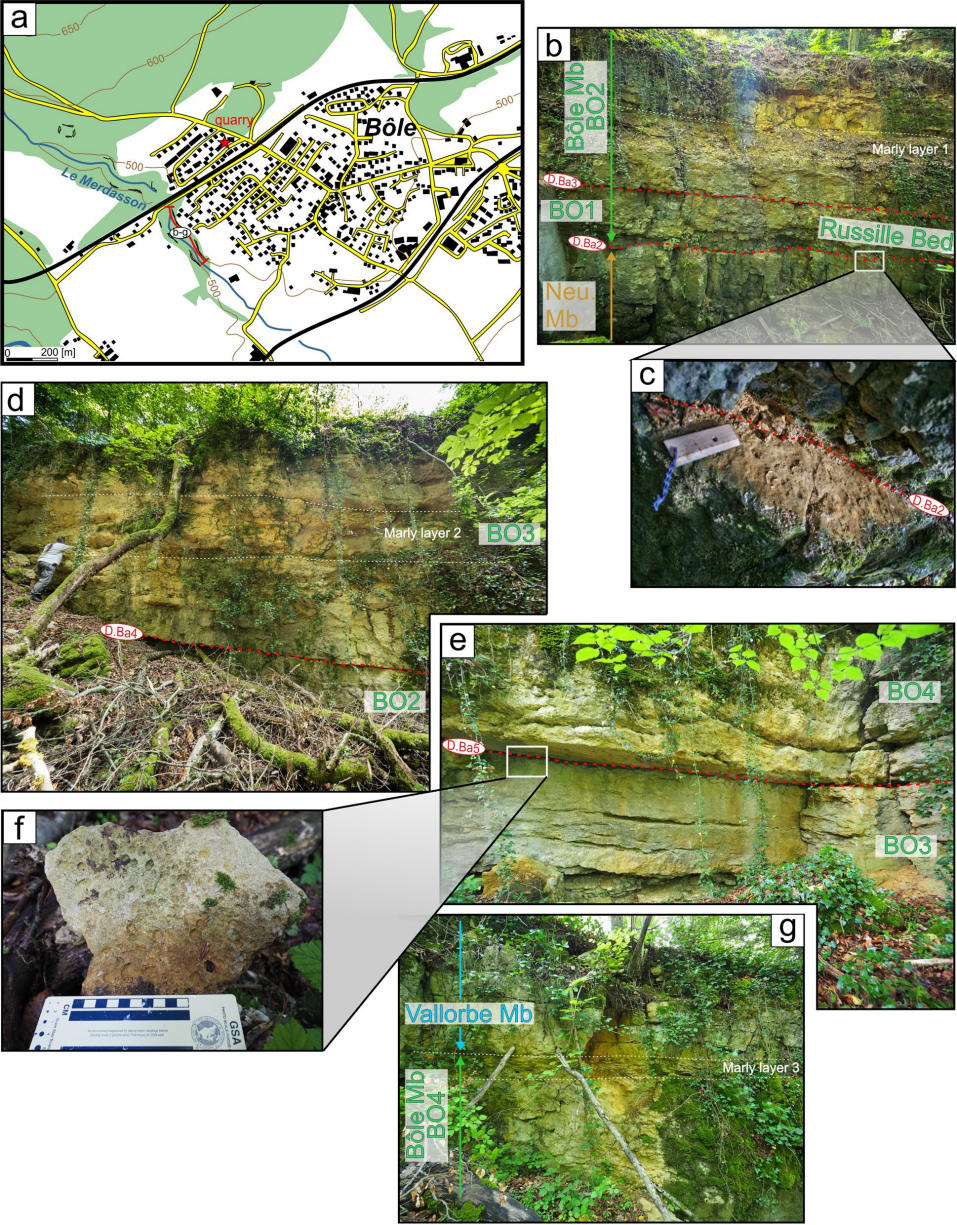
**a** Location map of the old Bôle quarry and of the type-section of the Bôle Member along the Merdasson stream (Neuchâtel, Switzerland). **b** Transition between the Neuchâtel and the Bôle members with the basal Marne de la Russille Bed, which is framed by discontinuity surfaces D.Ba2 and D.Ba3, and followed by the upper series of the Bôle Member. **c** Focus on the bioperforated and bioencrusted hardground on top of the Pierre jaune de Neuchâtel. **d** Middle part of the Bôle Member with the discontinuity surface D.Ba4 separating units 2 and 3. **e** Upper part of the Bôle Member with the discontinuity surface D.Ba5 separating units 3 and 4. **f** Focus on the bioperforated and bioencrusted hardground of D.Ba5. **g** Transition from the Bôle Member to the Vallorbe Member by the intermediary of a last marly intercalation

**Fig. 9:**
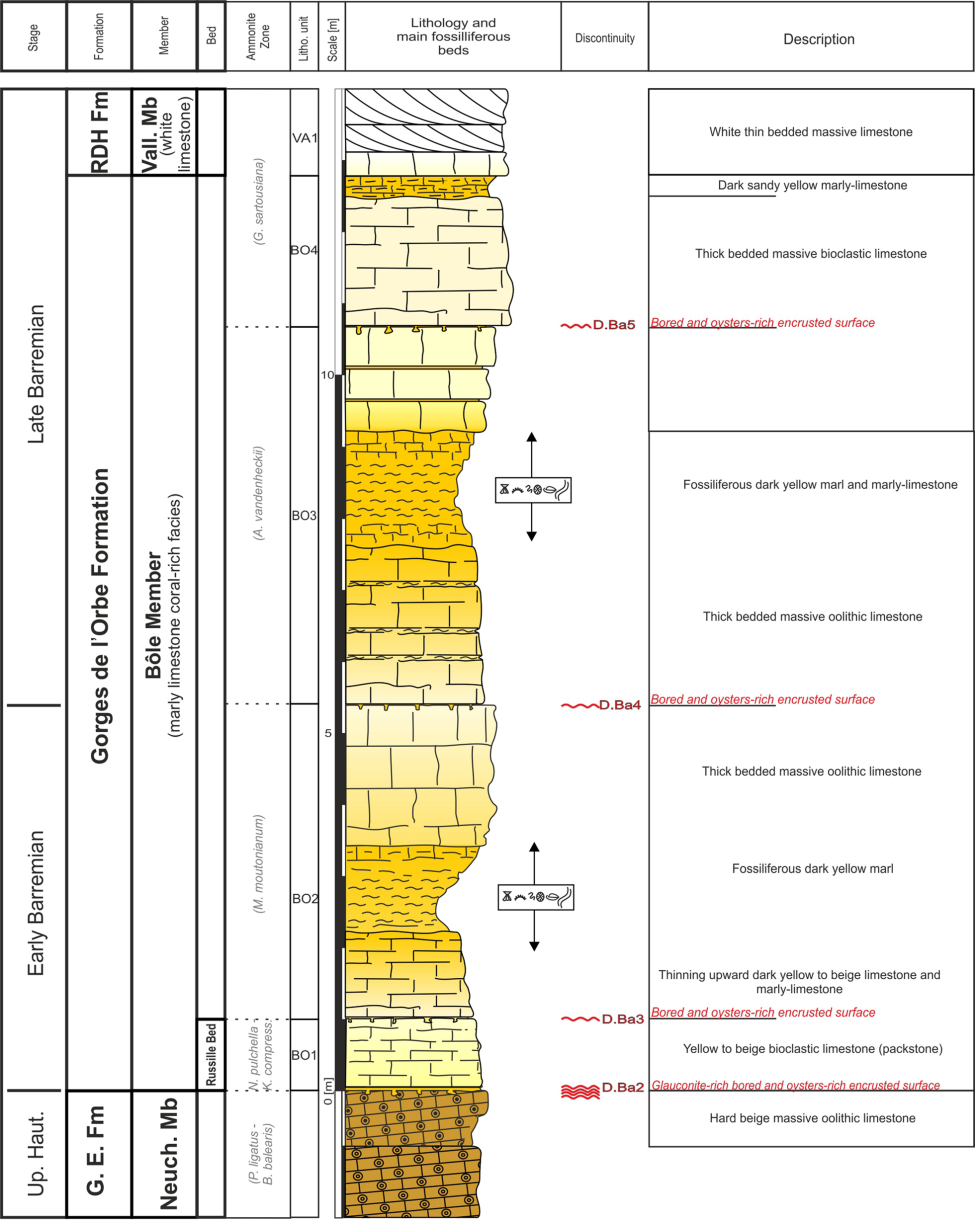
The Bôle Member type-section with age, litho- and biostratigraphy on the left side, and the main fossiliferous beds, discontinuity surfaces and description of the log on the right side

Although still accessible, the quarries of Bôle have recently been transformed into a residential area (Coordinates 2554′103/1202′180). Schardt and Dubois (1902, p. 277) report beautiful outcrops in the nearby Merdasson ravine at 350 m of the old quarries (Figs. 8, 9; Coordinates 2529′904/1201′883 to 2553′911/1201′907 system CH1903/LV03).

#### Thickness

The Bôle Member measures between 0 and 31 m depending of its tectonic position on topographic highs or depocenters. The maximum sedimentary record is encountered along the Vuache and Mollendruz faults, the thickest section being recorded at the Cul du Nozon section with a total thickness of 31 m.

#### Definition and boundaries

**Fig. 10:**
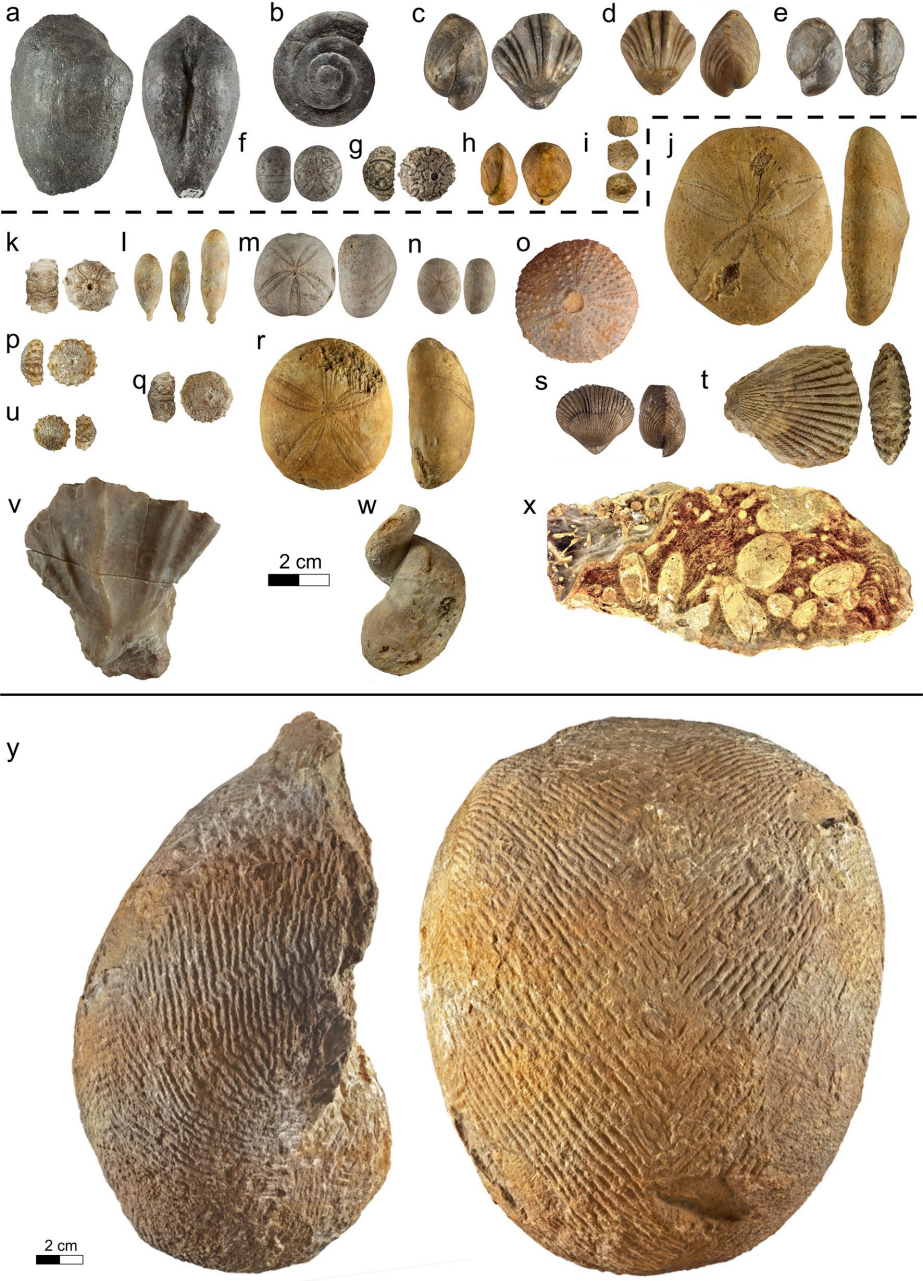
Fossils from the Bôle Member. **a**–**i** Marne de la Russille Bed, upper lower Barremian: **a** *Panopea neocomiensis* (Leymerie 1842), Eclépens, Vaud, Switzerland, MGL.107288 coll. A. Pictet. **b** *Pleurotomaria truncata* Pictet & Campiche 1863, Eclépens, Vaud, Switzerland, MGL.107289 coll. A. Pictet. **c** *Glosseudesia ebrodunensis* (de Loriol 1864), Eclépens, Vaud, Switzerland, MGL.107290 coll. A. Pictet. **d** *Glosseudesia ebrodunensis* (de Loriol 1864), La Russille, Vaud, Switzerland, MGL.107291 coll. A. Pictet. **e** *Loriolithyris russillensis* (de Loriol 1864), Eclépens, Vaud, Switzerland, MGL.107292 coll. A. Pictet. **f** *Globator cylindrica* (Gras 1848), Eclépens, Vaud, Switzerland, MGL.107293 coll. A. Pictet. **g** *Hyposalenia stellulatus* (Agassiz 1838), Eclépens, Vaud, Switzerland, MGL.107294 coll. A. Pictet. **h** *Sellithyris sella* (Sowerby 1823), La Russille, Vaud, Switzerland, MGL.107295 coll. A. Pictet. **i** *Codiopsis jaccardi* Cotteau 1866, Montcherand, Vaud, Switzerland, MGL.107296 coll. A. Pictet. **j**–**z** Upper series of the Bôle Member, uppermost lower to middle upper Barremian: **j** *Astrolampas productus* (Agassiz 1836), Eclépens, Vaud, Switzerland, MGL.107297 coll. A. Pictet. **k** *Pseudocidaris cluniferas* (Agassiz 1840), La Russille, Vaud, Switzerland, MGL.107303 coll. A. Pictet. **l** *Pseudocidaris cluniferas* (Agassiz 1840), Montcherand, Vaud, Switzerland, MGL.107298 coll. A. Pictet. **m** *Heteraster couloni* Agassiz 1839, globose morphotype, Mormont, Vaud, Switzerland, MGL.107299 coll. A. Pictet. **n** *Pygaulus desmoulinsi* Agassiz 1847, Musinens (Bellegarde-sur-Valserine), Ain, France, MGL.107300 coll. A. Pictet. **o** *Pseudodiademma jarccardi* Cotteau 1863, Vallorbe, Vaud, Switzerland, MHNG GEPI.5050 coll. F.-J. Pictet. **p** *Goniopygus peltatus* (Agassiz 1836), Musinens (Bellegarde-sur-Valserine), Ain, France, MGL.107301 coll. A. Pictet. **q** *Hyposalenia stellulatus* (Agassiz 1838), La Russille, Vaud, Switzerland, MGL.107302 coll. A. Pictet. **r** *Pygaulus morloti* Desor 1857, Montcherand, Vaud, Switzerland, MGL.107304 coll. A. Pictet. **s** *Lamellaerhynchia multicostata* Burri 1957, Montcherand, Vaud, Switzerland, MGL.107305 coll. A. Pictet. **t** *Pecten landronense* Loriol 1869, Alleveys, Vaud, Switzerland, MGL.21605 coll. GEOL_REG. **u** *Hyposalenia stellulatus* Loriol 1869, Montcherand, Vaud, Switzerland, MGL.107306 coll. A. Pictet. **v** *Agriopleura marticensis* (d’Orbigny 1850), Musinens (Bellegarde-sur-Valserine), Ain, France, MGL.107307 coll. A. Pictet. **w** *Requienia* sp., Les Ponts-de-Martel, Neuchâtel, Suisse, MGL.107308 coll. A. Pictet. **x** Bioperforated stromatoporoid, La Russille, Vaud, Suisse, MGL.107309 coll. A. Pictet. **y** *Eucymatoceras plicatum* (Fitton 1836), Mormont, Vaud, Suisse, MGL.21696 coll. GEOL_REG

The Bôle Member is defined by an alternation of extremely fossiliferous yellow marls and cream-coloured limestone units. When complete, the series is composed of 4 marl and limestone bundles, each topped by a perforated and encrusted hardground at the exception of the last bundle which grades upward to the “Urgonien blanc” facies by the intermediary of a strongly bioturbated bed. The member presents a lowermost marly level, the “Marnes de la Russille” in its original sense (Jaccard 1861, 1863, 1869), characterized by the rich faunal association of the bivalves *Panopea neocomiensis* (Fig. 10a), the brachiopods *Glosseudesia ebrodunensis* (de Loriol 1864) (Fig. 10c, d), *Loriolithyris russillensis* (de Loriol 1866) (Fig. 10e) and the echinids *Globator cylindrica* (Gras 1848) (Fig. 10f) and *Hyposalenia stellulatus* (Agassiz 1838) (Fig. 10g). The rest of the member, informally called “upper marls” by Jaccard (1869, p. 141), is distinguished by its numerous echinoids [*Astrolampas productus* (Agassiz 1836) (Fig. 10j), *Pseudocidaris clunifera* (Agassiz 1840) (Fig. 10k, l), *Heteraster couloni* (Agassiz 1839) (Fig. 10m), *Pseudodiademma jarccardi* Cotteau 1863 (Fig. 10o), *Goniopygus peltatus* (Agassiz 1838) (Fig. 10p), *Cidaris lardyi* Desor 1855, *Pygaulus morloti* (Desor 1857) (Fig. 10r), *Hyposalenia stellulatus* (Agassiz 1838) (Fig. 10q, u)], bivalves [*Pecten landronense* de Loriol 1869 (Fig. 10t)], brachiopods [*Lepidorhynchia dichotoma* Burri, 1956 (Fig. 10s)], gastropodes [*Natica (Amauropsis) bulimoides* (Deshayes in Leymerie 1842)], and cephalopods (*Eucymatoceras plicatum* Fitton 1836) (Fig. 10y) associated to abundant coral and stromatoporoid (Fig. 10x, de Loriol & Gilliéron (1869).

#### Subdivisions, geographic distribution and lateral variations

**Fig. 11:**
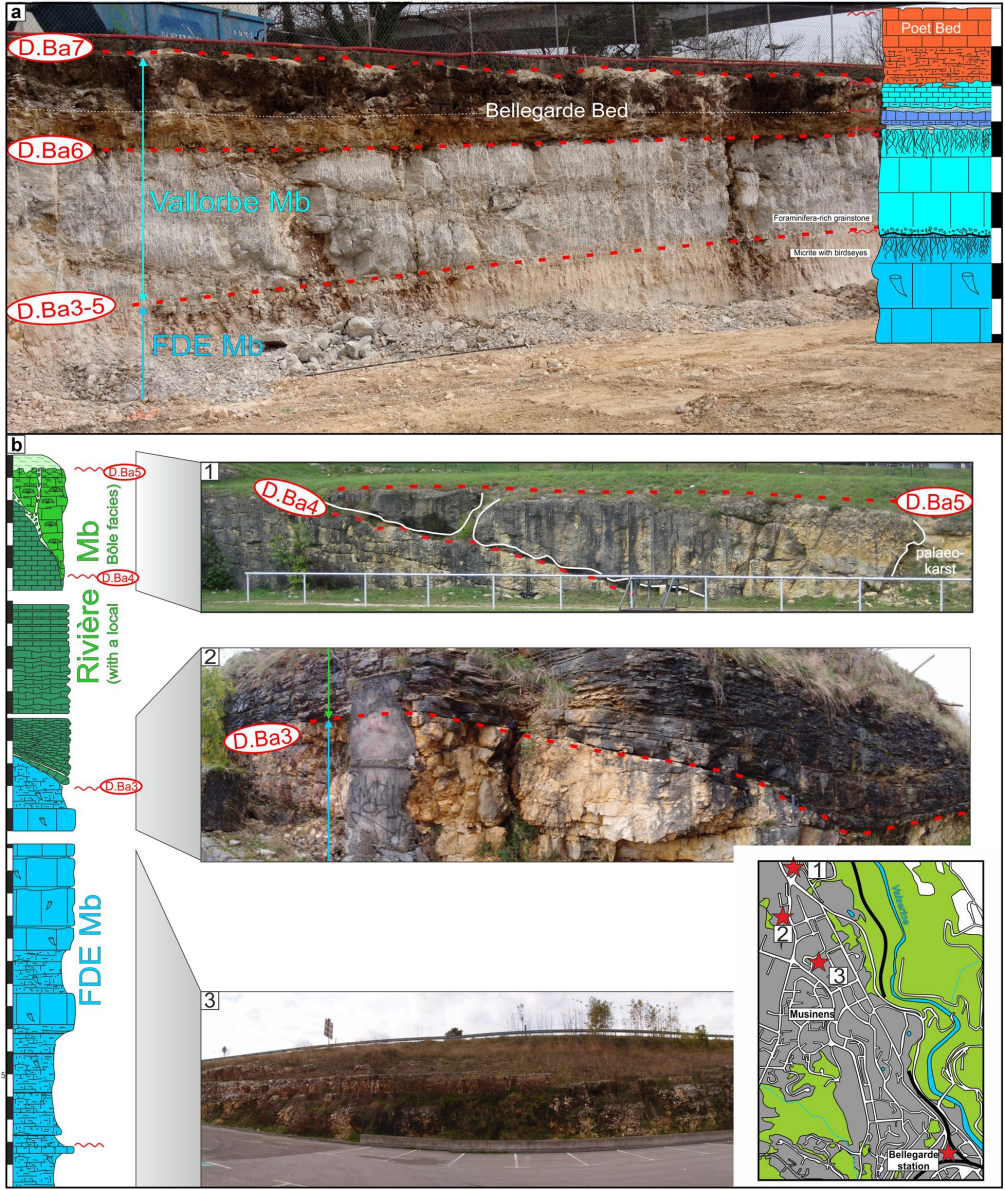
**a** Uppermost levels of the Rocher-des-Hirondelles Formation in the city-centre of Bellegarde-sur-Valserine (Ain, France) in a building foundation. It presents a reduced section with the staking of the Fort de l’Écluse Member and the Vallorbe Member with its Bellegarde Bed. Both members are separated by an angular and polyphased discontinuity surface D.Ba3-5 separating microporous and tight limestones from a foraminifera-rich grainstone. D.Ba3-5 and D.Ba7 discontinuity surfaces cover more than half of the Barremian stage. **b** Section and photographies of the nested palaeovalleys of Musinens in the north eastern area of Bellegarde-sur-Valserine, filled with the Bôle Member. This thick sedimentary succession pinches laterally to become D.Ba3-5

The member is mostly restricted to the Central Jura between St-Georges to the SW and Le Landeron to the NE. The Marne de la Russille Bed, like the Pont des Pierres Bed, is recorded only sporadically, appearing and disappearing regularly along the SW–NE axis, mostly restricted in lowered compartments of tilted blocks. The largest occurrence is a vast lens cropping out between Bretonnières and La Russille. Both extremities are characterized by a 0 to 30 cm thick fossils-rich conglomerate associated to abundant glauconite and phosphate-coated pebbles. In the middle of this lens, in the Gorges de l’Orbe canyon, the Marne de la Russille Bed is recorded by a 1-m-thick marly subunit separated from the upper part of the Bôle Member by a perforated and encrusted hardground (D. Ba3) on top of a fossils conglomerate associated to glauconite. The Marne de la Russille Bed becomes more constant and thicker toward the eastern Central Jura like in Le Mail section (Coordinates 2562′852/1205′248 to 2563′012/1205′506 system CH1903/LV03), in the city of Neuchâtel, where it reaches its maximum thickness with 5 m. In many other sections of the Neuchâtel area, the Bôle Member directly starts on top of the Grand Essert Formation by its upper series. The Bôle Member laterally passes south-westward to the shallower Rivière Member from the Meridional Jura as well as in rare places of the westward Central Jura (see Sect. 4.2, Schardt, 1892). Occasional south-westward occurrence of the Bôle type-facies can be observed inside the Rivière Member like at Musinens (Bellegarde-sur-Valserine, Fig. 11, coordinates 5.81633 m E/46.1232 m N system WGS84, projection UTM, zone 32 T), intercalated between the Fort de l’Écluse and the Vallorbe members. This local recurrence of the Bôle facies is here represented by more than 15 m thick series which fills nested palaeovalleys by the intermediary of the strongly erosive and angular discontinuity surfaces D.Ba3, D.Ba4 and D.Ba5 which are also bored and bioencrusted. First sedimentary sequence above D.Ba3 is characterized by thick tidalites (Fig. 11b2). Second sedimentary sequence above D.Ba4 is composed of coral-rich limestones strongly karstified and vertically crossed by karstic wells which reach the discontinuity surface D.Ba4 along which it develops obliquely (Fig. 11b1). Third sedimentary sequence above D.Ba5 was mostly observed as typical Bôle marls with abundant sponges, echinoids, bivalves, and gastropods filling the below karstic network. These nested palaeovalleys laterally disappear in a single discontinuity surface D.Ba3-5 separating the Fort de l’Écluse and the Vallorbe members like in the Bellegarde-sur-Valserine city centre (Fig. 11a).

#### Biostratigraphic data and age

The Marne de la Russille Bed delivered a millimetric ammonite with an ornamentation quite typical for an Holcodiscidae (*Pseudometahoplites* sp. juv.?) possibly from the late early Barremian (see De Kaenel et al., 2020). The ostracod association of the Bôle Member is listed in Clavel et al., (1994), Mojon et al. (2013) and Mojon in Pasquier et al. (2013). The member is marked by abundant *Schuleridea* gr. *rhomboidalis* Neale 1960, *Cytherella* gr. *parallela* Reuss 1846, *Bairdoppilata luminosa* Kuznetsova 1961, *Cytherelloida* spp., *Strigosocythere strigosa* (Grosdidier 1964), *Schulderidea* spp., *Bairdopilata* sp., *Rehacythereis geometrica* (Damotte & Grosdidier 1963), B. sp., and *Neocythere (Centrocythre)* gr. *gottisi* Damotte & Grosdidier 1963 while *Protocythere triplicata* is absent. This modified association and the apparition in the higher levels of *Strigosocythere chalilovi* (Kuznetsova 1961) also point to a late early to early late Barremian age (Babinot & Collin 2011; De Kaenel et al., 2020; Lukeneder, 2010; Mojon in Eichenberger et al., 2020). The brachiopods and echinoids association are typically Barremian in age (e.g. Masse & Humbert, 1976). New nannoflora studies in the Eclépens and La Sarraz sections were performed by De Kaenel et al. (2020). The *Glosseudesia ebrodunensis*-rich Marne de la Russille Bed is placed in the LK20B nannofossil Zone, corresponding to the lower *K. compressissima* ammonite Zone with the acme of *C. margerelii* Noël 1965. Younger levels of the Bôle member are attributed to the Boreal LK19-LK18 and Tethyan NC5D nannofossil Zones, corresponding to the *M. moutonianum* to lower *G. sartousiana* ammonite zones.

## Rocher des Hirondelles Formation (RDH Fm)

### Definition

The Urgonian stage was introduced by d'Orbigny (1850) at the type-locality of Orgon (Bouches-du-Rhône, France) as a geological stage to replace the Upper Neocomian characterized by rudistic white limestones (Rat, 1983). This term is actually commonly used as a facies in reference to any Cretaceous limestone which contains rudists, a highly asymmetric bivalve which is characteristic of many Cretaceous shallow-water photozoan carbonate platforms (Hunt, 1992).

In the Jura Mountains, de Tribolet (1856, 1857, 1859) designated as “Couches du Mormont/Mauremont” the white rudists-bearing limestones described by Itier (1842, 1843) and Favre (1843). In the same period, Marcou (1859) proposed the “Calcaires de Noirevaux-dessus”, and Desor and Gressly (1859) the ‘‘Urgonien supérieur” (Fig. 3). Marcou’s denomination was rejected by de Tribolet (1859) and by Jaccard (1893) because the type locality is represented by outcrops of very poor quality. For the same reason, we can also reject the “Couches du Mormont/Mauremont” of de Tribolet (1856, 1857, 1859). Furthermore, both localities are poorly chosen because representing only a minor part of the white rudists-bearing limestones. Custer (1928) introduced the facies denomination ‘‘Urgonien blanc’’ which remained in use for some decades (Fig. 3). Conrad (1969) introduced a “Membre des calcaires urgoniens inférieurs” and a “Membre des calcaires urgoniens supérieurs” to name both urgonian cliffs of the Meridional Jura. Although their stratigraphical correspondence to our formation and members, these terms were not retained since both “Membres des calcaires urgoniens” do not respect the rules of the International Stratigraphic Commission (ISC). Strasser et. al. (2016) proposed the Vallorbe Formation in the same spirit than de Tribolet, Marcou and Conrad (Fig. 3). Nevertheless, Strasser et. al. (2016, p. 12) considered the “Urgonien blanc” facies as isochronous through the Jura Mountains. New field observations and dating allow to question these isochronies of the Early Cretaceous formations and seems to show strongly diachronous facies (lithostratigraphic units) passing laterally from one to the other. Furthermore, the white Urgonian facies seems arranged in successive lenses in forced progradation, where the “Vallorbe Formation” of Strasser et. al. (2016) only represents a single sedimentary lens mostly restricted to the Central Jura. For these reasons, we propose to introduce a new formation, the Rocher-des-Hirondelles Formation (Fig. 3), which covers the whole succession of the “Urgonien blanc” facies.

#### Type locality

**Fig. 12:**
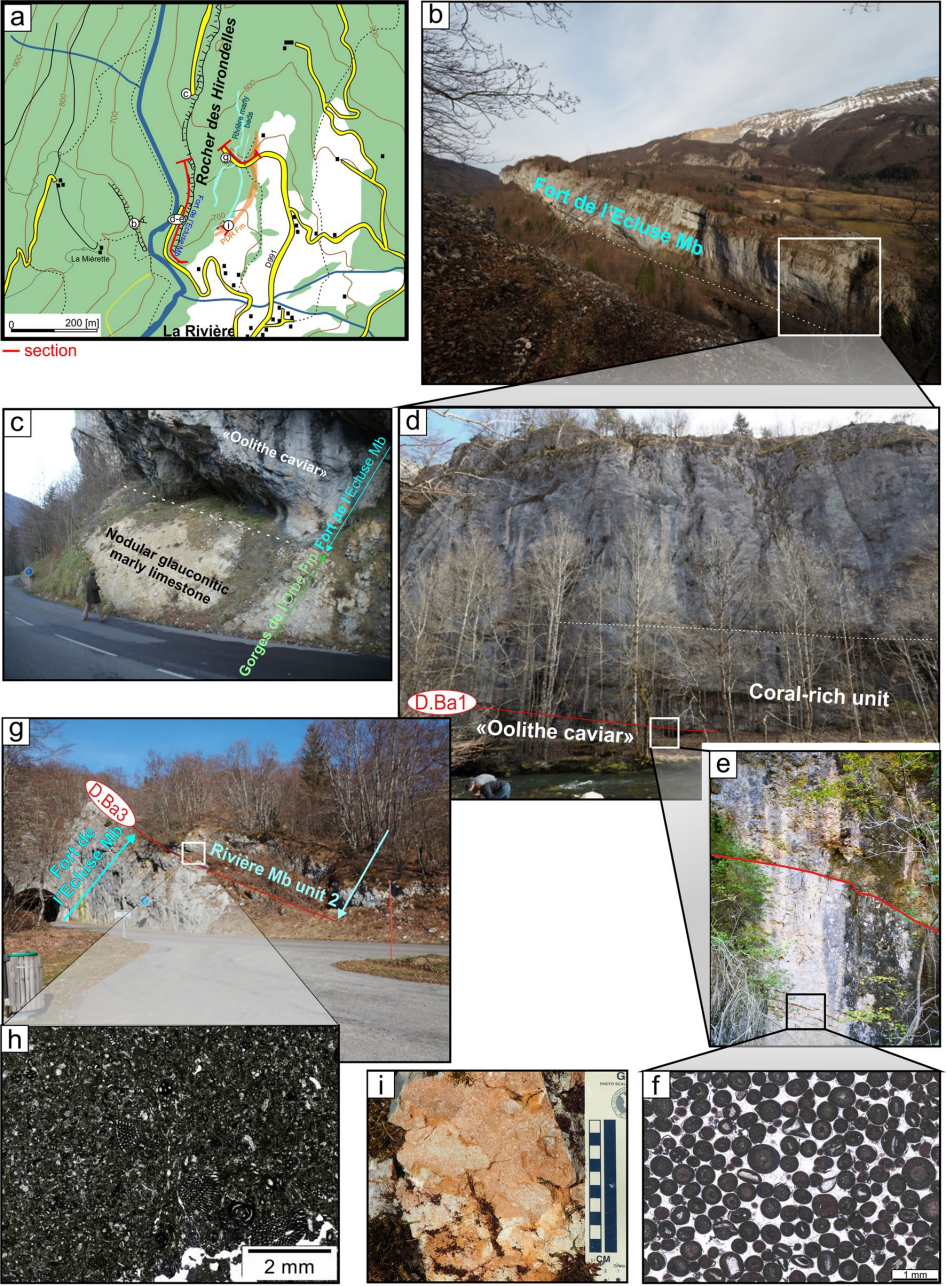
**a** Location map of the Rocher des Hirondelles Formation at the Rocher des Hirondelles type-sections (Ain, France) and field-photographies. **b** General view of the Fort de l’Écluse limestone cliff. **c** Boundary between the Cul du Nozon Bed (GDO Fm) and the “Oolithe caviar” (Fort de l’Écluse Member) showing a progressive transition from marls to oolitic carbonates, northern exit of the Rocher des Hirondelles tunnel. **d** View of the Fort de l’Écluse Member at the type section with the 3 carbonated sedimentary units with from bottom to top, the “Oolithe caviar”, the coral-rich unit, and the main limestone mass. **e** Focus on the bored and bioencrusted discontinuity surface D.Ba1 between the “Oolithe caviar”, the coral-rich unit. **f** Thin section in the oolitic grainstone of the “Oolithe caviar” representing hydraulic-dune environments. **g** Boundary between the main mass (Fort de l’Écluse Member) and the Rivière Member separated by the bored et bioencrusted discontinuity surface D.Ba3 at the southern exit of the Rocher des Hirondelles tunnel. **h** Thin section in the *Orbitolina*-rich, micritic, open-sea, limestone of the Rivière Member. **i** View of the orange limestone of the Poet Bed below the southern exit of the Rocher des Hirondelles tunnel

**Fig. 13:**
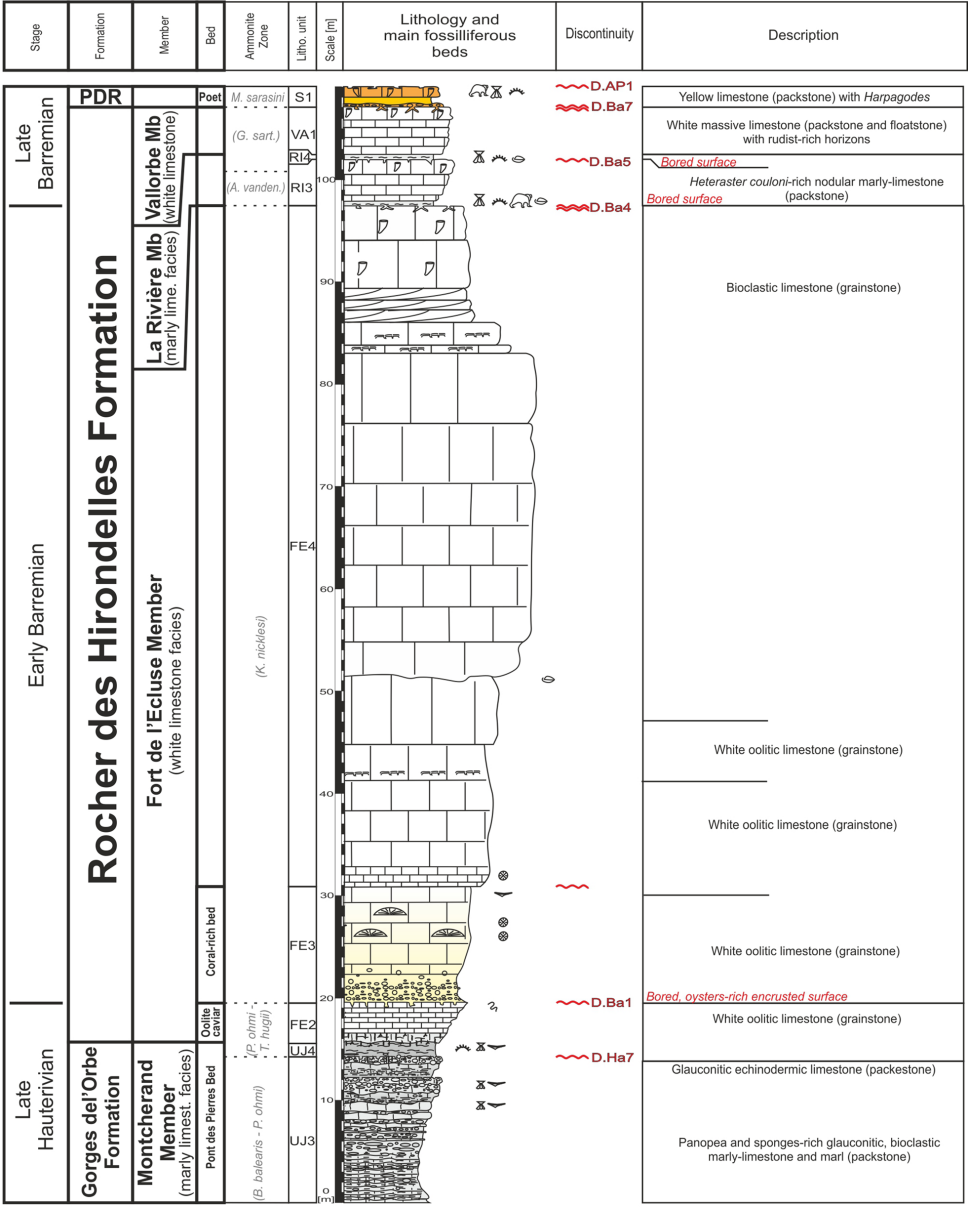
The Rocher des Hirondelles Formation type-section with age, litho- and biostratigraphy on the left side, and the main fossiliferous beds, discontinuity surfaces and description of the log on the right side

The type locality is situated in the Valserine valley near the La Rivière locality, 15 km north-eastward from Bellegarde-sur-Valserine (Fig. 12). The section (Fig. 13) has been relogged, in three parts with: (i) the lower urgonian unit along the cliff bordering the Valserine river (Coordinates 5.89233 m E/46.24733 m N to 5.89228 m E/46.24301 m N system WGS84, projection UTM, zone 31 T); (ii) the Rivière Member and the upper Urgonian unit in front of the entry of the tunnel (Coordinates 5.89415 m E/46.24662 m N to 5.89592 m E/46.24627 m N system WGS84, projection UTM, zone 31 T); (iii) below the tunnel, at the forest border for the Poet Bed from the Perte-du-Rhône Formation (Pictet et al., 2016a; Coordinates 5.89573 m E/46.24501 m N system WGS84, projection UTM, zone 31 T). The section of the Rocher-des-Hirondelles was described in detail by Conrad (1968, 1969), Viéban (1983) and Blanc-Alétru (1995).

#### Thickness

RDH Fm presents its maximum observed thickness in the Meridional Jura with up to approximatively 180 m in the Bellegarde-sur-Valserine depocenter. Nevertheless, strong laterally thickness and facies changes are observed on only few kilometres following a succession of depocenters and topographic highs delimited by syn-sedimentary faults.

#### Definition, subdivisions and boundaries

In the Meridional Jura, the RDH Fm overlies the Montcherand Member, while in the Central Jura, it tops the Bôle Member. The lithology consists of thick massive beds of white or light yellowish limestone commonly containing rudist debris. The microfacies is usually a bioclastic limestone, composed of grainstones, packstones, and wackestones. Although exceptional, glauconite grains are present in outer platform sediments like at the base of the formation (e.g. La Chambotte section) or on its distal borders (e.g. Serrières Bed in Le Landeron-Combes area). The quartz, which is usually absent, can be strongly enriched to the top of the formation toward the Meridional Jura (e.g. Bellegarde Bed in the type locality, and the Lower Orbitolina Beds of La Chambotte section). Microfossils include abundant large foraminifera as orbitolinids and miliolids, dasycladalean and charophyte algae, all typical of a shallow, inner platform environment with marine to lacustrine deposits. The formation is subdivided in three successive members with: (i) at the bottom, the Fort de l'Écluse Member (= Membre des calcaires urgoniens inférieur of Conrad, 1969); (ii) in the middle, the Rivière Member; (iii) at the top, the Vallorbe Member (= Membre des calcaires urgoniens supérieurs of Conrad, 1969), comprising the Bellegarde and the Serrières beds at its top. The formation is generally topped by the Poet Bed of the Perte-du-Rhône Formation (Conrad, 1968, 1969; Pictet et al., 2016a, 2016b, 2019; Tsytovitch 1910).

#### Geographic distribution and lateral equivalents

The RDH Fm is restricted to the Jura Domain. It passes eastward to the GDO Fm (e.g. Brasserie de Morteau and Combes-Le Landeron sections), southeastward to the Helvetic Schrattenkalk Formation (Studer, 1834) and southward to the Subalpine Urgonian Formation (Arnaud-Vanneau & Arnaud 1990).

#### Biostratigraphic data and age

The RDH Fm was historically reported to the Barremian stage by Baumberger (1901) who parallelized the Urgonien blanc with the Calcaires urgoniens of the southern Subalpine chains, dated by ammonites from the Barremian by Paquier (1898). But this correlative dating is subject to strong controversies opposing two age-models referring on benthic microfossil scales with, a model supporting a late Hauterivian–earliest Barremian age (Clavel et al., 2007, 2013, 2014; Masse et al., 1998), and another model claiming a late Barremian age (Arnaud et al., 1998; Blanc-Alétru, 1995; Godet et al. 2010, 2012). In front of such age divergence, the calibration and the facies dependence of benthic microfossils is highly questionable. To avoid this problem, biostratigraphic ages detailed in the next chapters are mostly referring on: ostracods; echinoids; rudist shells; sections correlations; sequence stratigraphic interpretations; rare cephalopod findings allowing to locally improve or confirm the dating. The basal sedimentary sequence, comprising the Cul du Nozon Bed, delivered a latest Hauterivian to earliest Barremian fauna (see Sect. 3.1), and the Poet Bed of the Perte-du-Rhône Formation above delivered the ammonite *Martelites* sp. juv. from the latest Barremian *M. sarasini* Subzone (Pictet et al., 2019). This framing dating suggest that in the Meridional Jura the RDH Fm covers series from the latest Hauterivian to the latest Barremian (*I. giraudi* Zone?), while in the Central Jura the formation would be restricted to the latest Barremian (*G. sartousiana* Zone, Godet et al., 2011).

### Fort de l'Écluse Member

#### Type locality

**Fig. 14:**
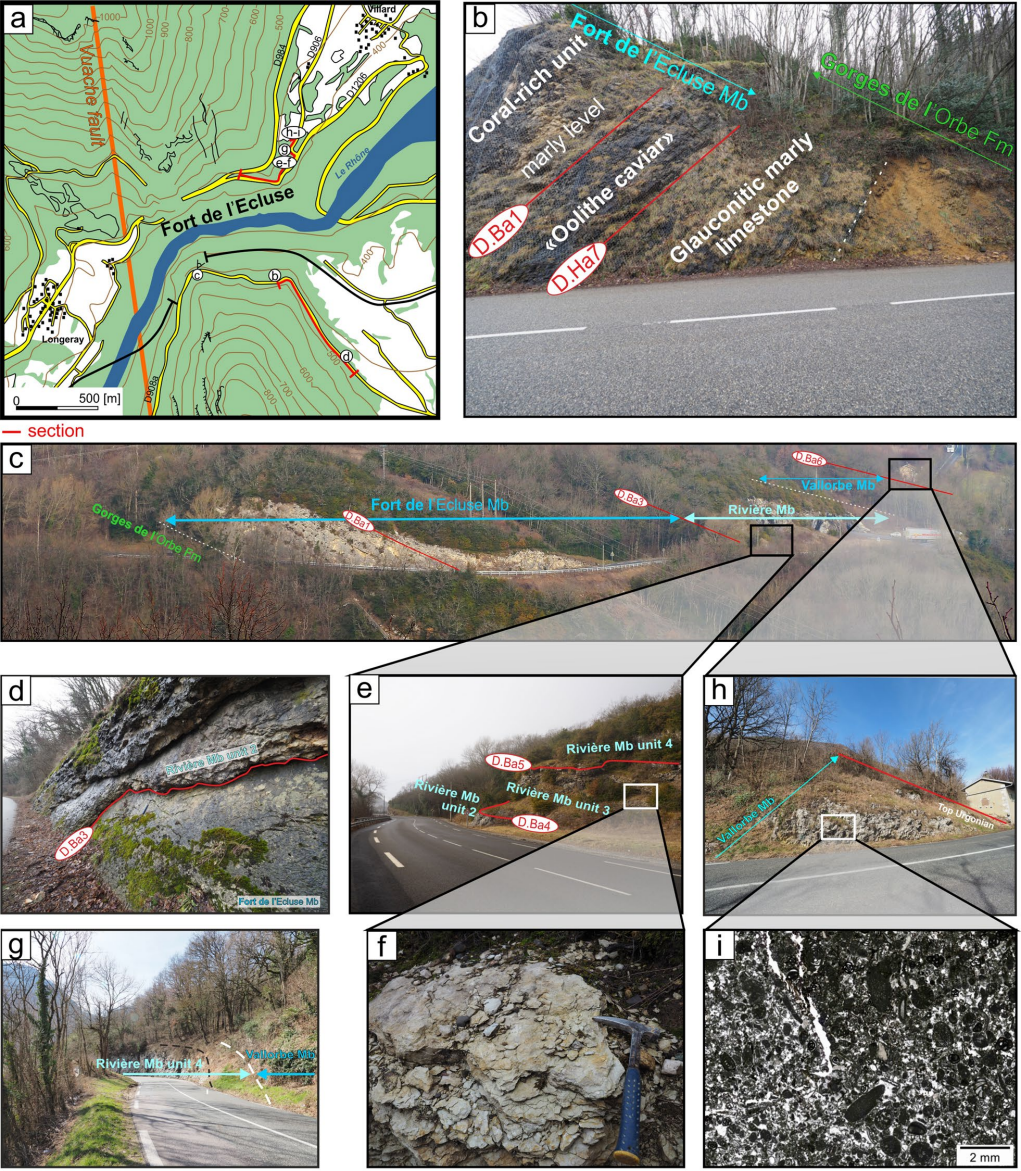
**a** Location map of the Fort de l’Écluse Member type-sections in the locality of the same name (Ain and Haute-Savoie, France). **b** Transition from the GDO Fm to the RDH Fm. The Cul du Nozon Bed is here missing and the “Oolithe caviar” directly tops the Pont des Pierres Bed by the intermediary of the discontinuity surface D.Ha7. The “Oolithe caviar” is separated from the Coral-rich unit by the intermediary of the discontinuity surface D.Ba1 and its above marly level. **c** General view of the RDH Formation from the opposite side of the Fort de l’Écluse. **d** Focus on the bored et bioencrusted discontinuity surface D.Ba3 along the road D908a. **e** Focus on the Rivière Member along the road D1206 near the old customs post. **f** Focus on the *Heteraster couloni*-rich marly-limestone of the Rivière Member. **g** Transition from the Rivière Member to the Vallorbe Member near the old customs post. **h** Top of the Vallorbe Member which is epikarstified. **i** Thin section in the foraminifer-rich lagoonal facies of the Vallorbe Member

**Fig. 15:**
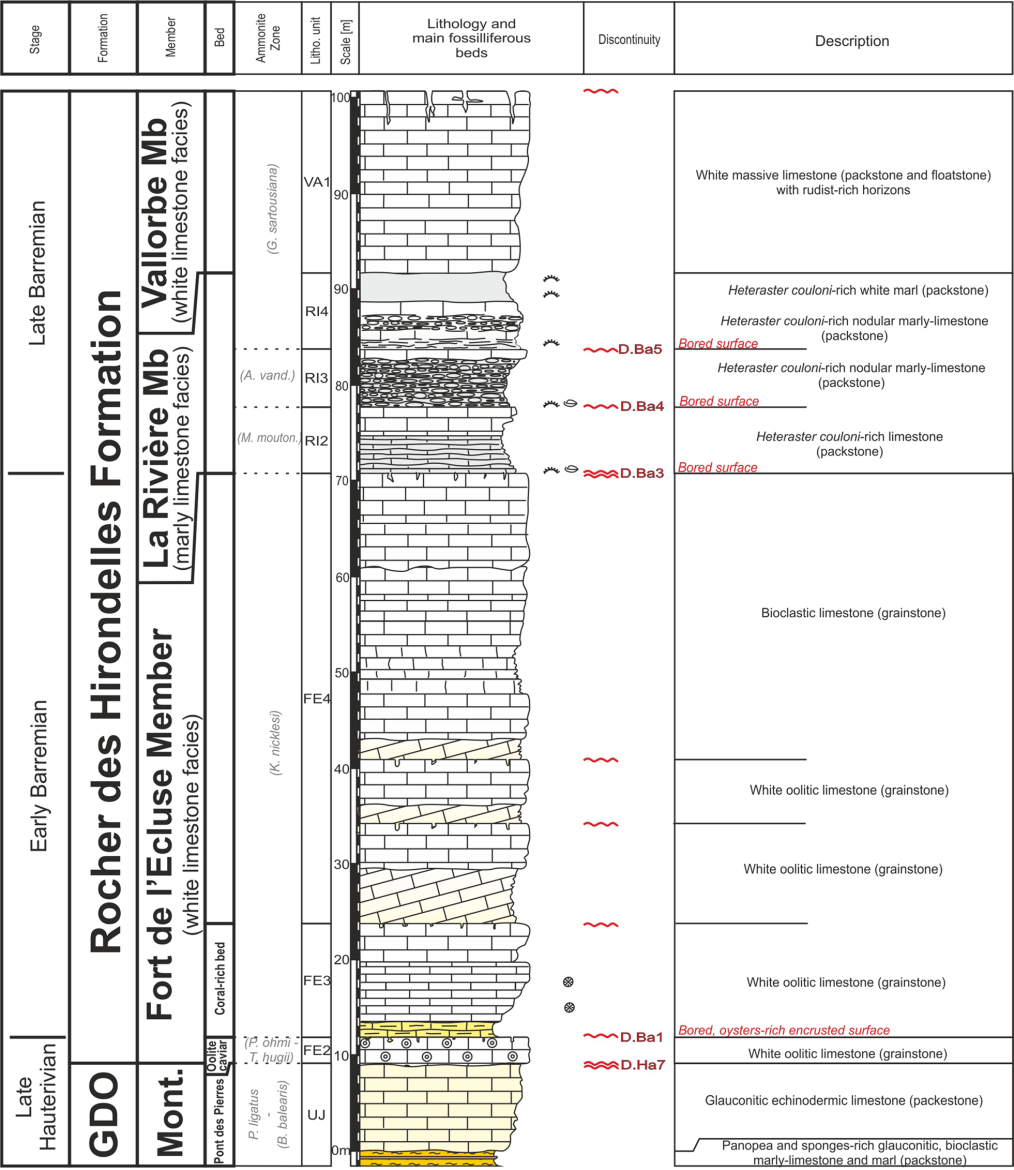
The Fort de l’Écluse Member type-section with age, litho- and biostratigraphy on the left side, and the main fossiliferous beds, discontinuity surfaces and description of the log on the right side. The Fort de l’Écluse Member was logged along the road D908a, while the Rivière and Vallorbe Member were logged on the opposite side of the Rhône river along the roads D1206 and D906

The Fort de l'Écluse type section crops out at the Fort de l'Écluse (Ain Department, France; Figs. 14, 15) and presents two complementary sections well exposed along two departmental roads. The section cropping out along the D.908a allows a good observation of the Fort de l'Écluse Member comprised between the underlying GDO Fm and the overlying Rivière Member (Fig. 14; Coordinates 5.89923 m E/46.11707 m N to 5.90427 m E/46.11346 m N system WGS84, projection UTM, zone 31 T). A good outcrop of the Rivière and Vallorbe members is observable on the opposite slope along the road D.1206 (Fig. 14E–G; Coordinates 5.89711 m E/46.12244 m N to 5.90168 m E/46.12653 m N system WGS84, projection UTM, zone 31 T).

#### Thickness

The Fort de l'Écluse Member presents its maximum observed thickness in the Meridional Jura with around 130 m in the Bellegarde-sur-Valserine depocenter. In addition of strong lateral thickness and facies changes observed on only a few kilometres, the member progressively thins and disappears in direction of the Central Jura with its more distal occurrence around the Vallorbe area.

#### Definition and boundaries

The Fort de l'Écluse Member is proposed for the first photozoan carbonate platform unit framed by the GDO Fm and the Rivière Member. The lithology consists of decimetric to plurimetric thick massive beds of light yellowish to white limestone currently presenting cross-bedded oolitic or bioclastic layers at its base and/or near its top. More generally, the limestone is arranged in a massif cliff or in an alternation of microporous and tight limestones with rudist debris (Richard et al., 2007; Volery et al., 2010) and root-traces. The member ends by a strongly angular perforated surface (D. Ba2–D. Ba3), locally presenting palaeovalley topographies.

#### Subdivisions, geographic distribution and lateral variations

No subdivision was introduced but three main limestone units were observed. The lowermost unit, which is an oolitic grainstone, is informally called “oolithe caviar” (e.g. Charollais et al., 2013b) and does not extend further north-eastward than the La Cure area in the French Jura Department. The “oolithe caviar” ends with a prominent bored and encrusted hardground (D.Ba1). The median unit is composed of coral-rich limestones which extend eastward up to St Georges in the Vaud canton. The upper unit is characterized by rudist-rich limestones extending eastward up to St Georges-Morand area. This facies of inner platform environments is characterised by the cyclic development of a freshwater table (Richard et al., 2007; Volery et al., 2010).

#### Biostratigraphic data and age

No direct dating is possible since only non-age-diagnostic nautilids were collected, and the absence of marly beds prevented any ostracod sampling. Nevertheless, echinoids collected in the underlying Cul du Nozon Bed point to a latest Hauterivian age for the onset of the “oolithe caviar” in the Chambotte area, and to an earliest Barremian age in the Valserine valley. The overlying Rivière and Bôle members delivered a late early to early late Barremian fauna (see below Sect. 4.2). The framing ages point to a time interval ranging from the latest Hauterivian to the late early Barremian for the deposition of the Fort de l’Écluse Member.

### Rivière Member

The first description of this lithostratigraphic unit was given by Favre (1843) who described, in the upper third of the Urgonian limestone of the Mt Salève (Haute-Savoie, France), a *Terebratula*-rich layer, “La couche à Térébratules” (see also Favre 1867). De Loriol in Favre (1867) described the rich fauna of this layer with echinoids as *Pseudocidaris clunifera* (Agassiz 1840) and *Goniopygus peltatus* (Agassiz 1838), and the brachiopod as *Loriolithyris russillensis* (de Loriol 1866) usually more typical from the Bôle Member. Itier (1843) noted in the nearby Mont Vuache the presence of nodular limestones, inside the white limestones of the Neocomien supérieur (= Urgonian facies), extremely rich in echinoids related to “*Spatangus retusus*”. In its masterful study of the Meridional Jura, Schardt (1892) recognized an up to 13 m thick series of marls and limestones rich in the echinoid *Heteraster couloni*, in the upper part of his “Urgonien I”. Later, Conrad (1969) observed a marly-limestone unit, located on top of his “Membre des calcaires urgoniens inférieurs”, which he named “Membre des Calcaires marneux de la Rivière”.

#### Type locality

**Fig. 16:**
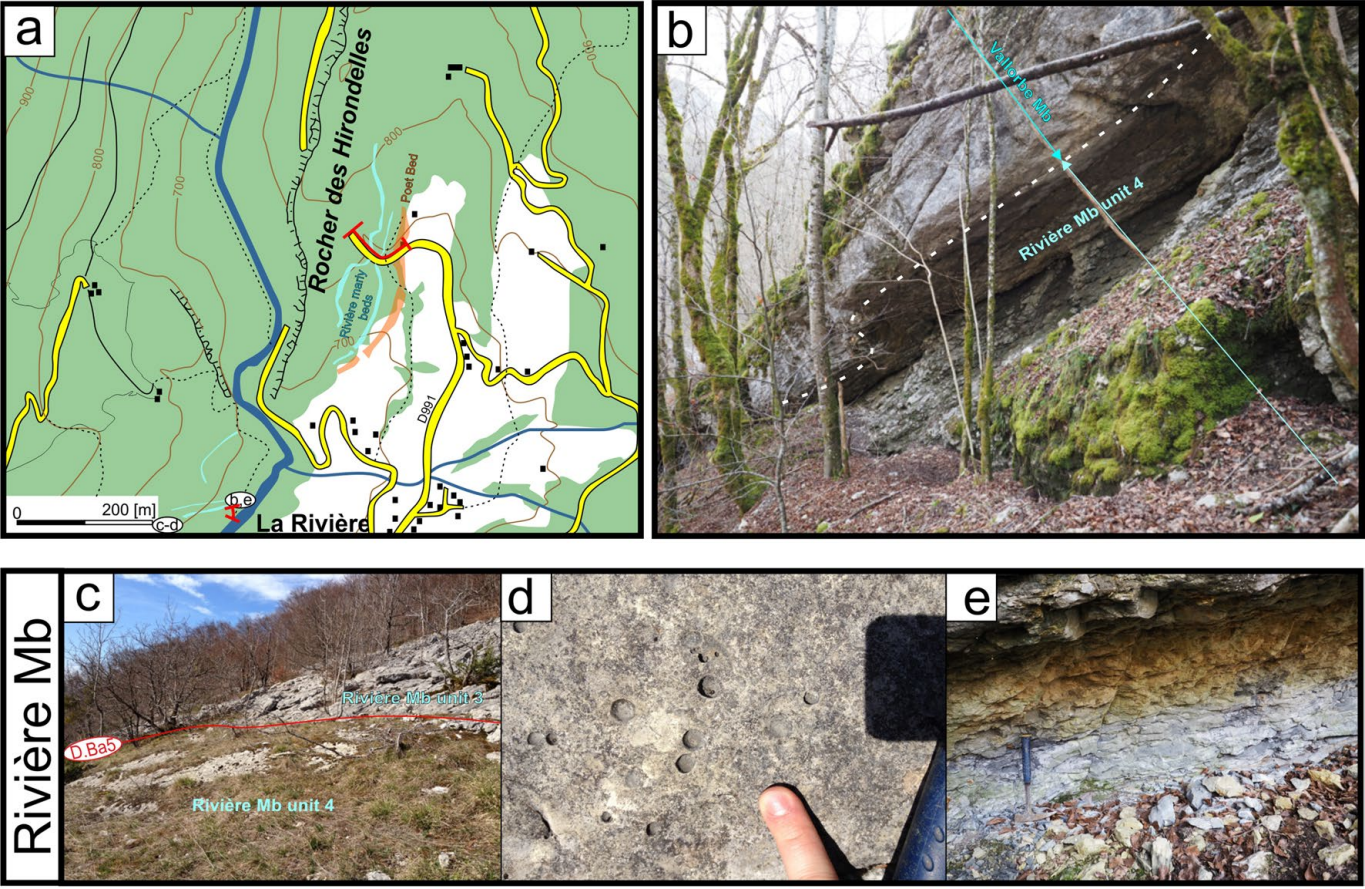
**a** Location map of the type-sections of the Rivière Member in the locality of the same name (Ain, France) and field-photographies. **b** General view of the La Rivière type section and the median and higher marly intercalations below the Vallorbe Member. **c** View of the discontinuity surface D.Ba5 separating the lower and median sequences of the Rivière Member. **d** Focus on the bioperforated hardground D.Ba4. **e** Focus on the *Heteraster couloni*-rich marl of the median intercalation

**Fig. 17:**
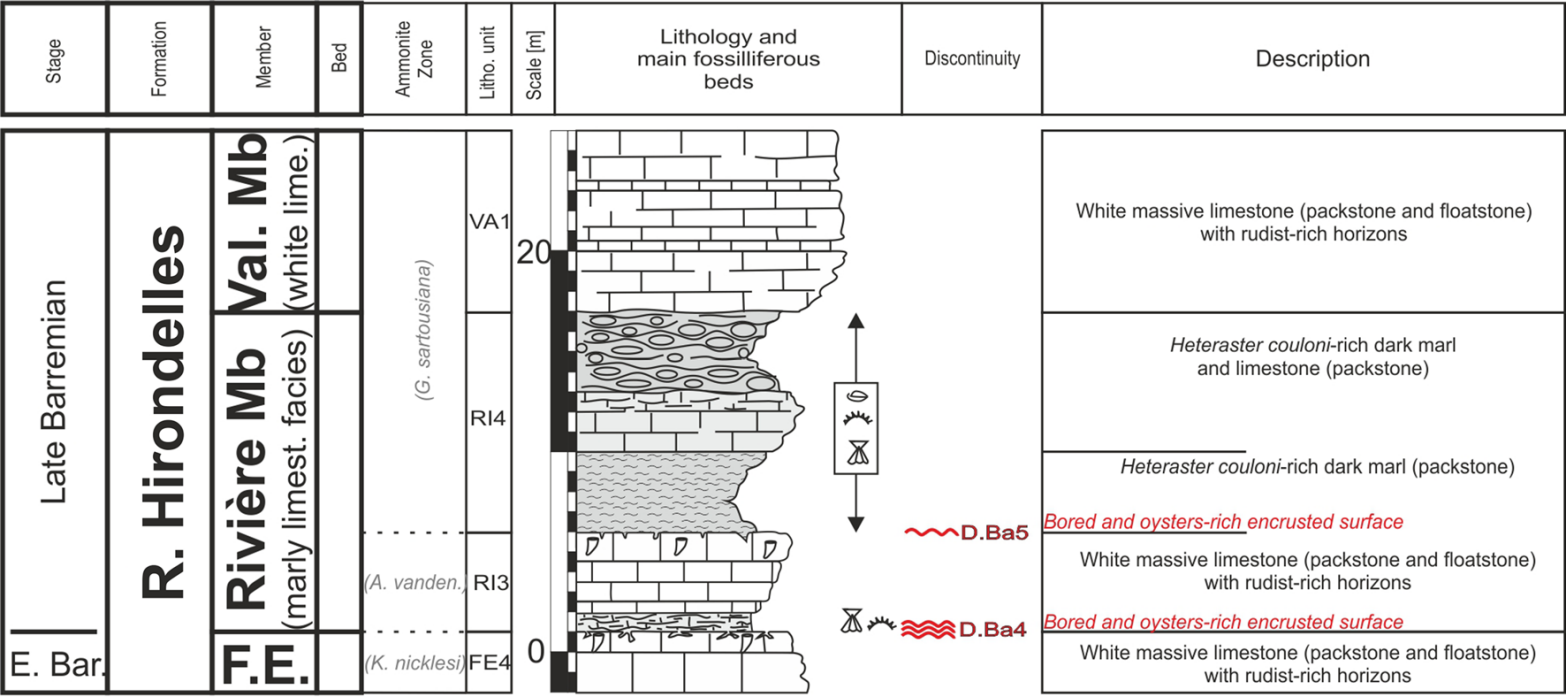
The Rivière Member type-section with age, litho- and biostratigraphy on the left side, and the main fossiliferous beds, discontinuity surfaces and description of the log on the right side

The Rivière Member was defined by Conrad (1969) at a small cliff, which crops out in front of the La Rivière village in the Valserine valley (Ain Department, France; Fig. 16). The section (Fig. 17) is situated on the right side of the river along the path descending from La Miérette (Coordinates to 5.89168 m E/46.24239 m N to 5.89058 m E/46.240623 m N system WGS84, projection UTM, zone 31 T).

#### Parastratotype

Fort de l'Écluse (Fig. 14c, e); Coordinates 5.90043 m E/46.12274 m N to 5.90135 m E/46.12490 m N (system WGS84, projection UTM, zone 31 T). This outcrop represents the most developed section of this facies with a ~ 21 m thick series.

#### Thickness

The Rivière Member, recorded by numerous sedimentary lenses of various thickness, presents its maximum observed thickness in the Meridional Jura (e.g. Fort de l'Écluse section).

#### Definition and boundaries

**Fig. 18:**
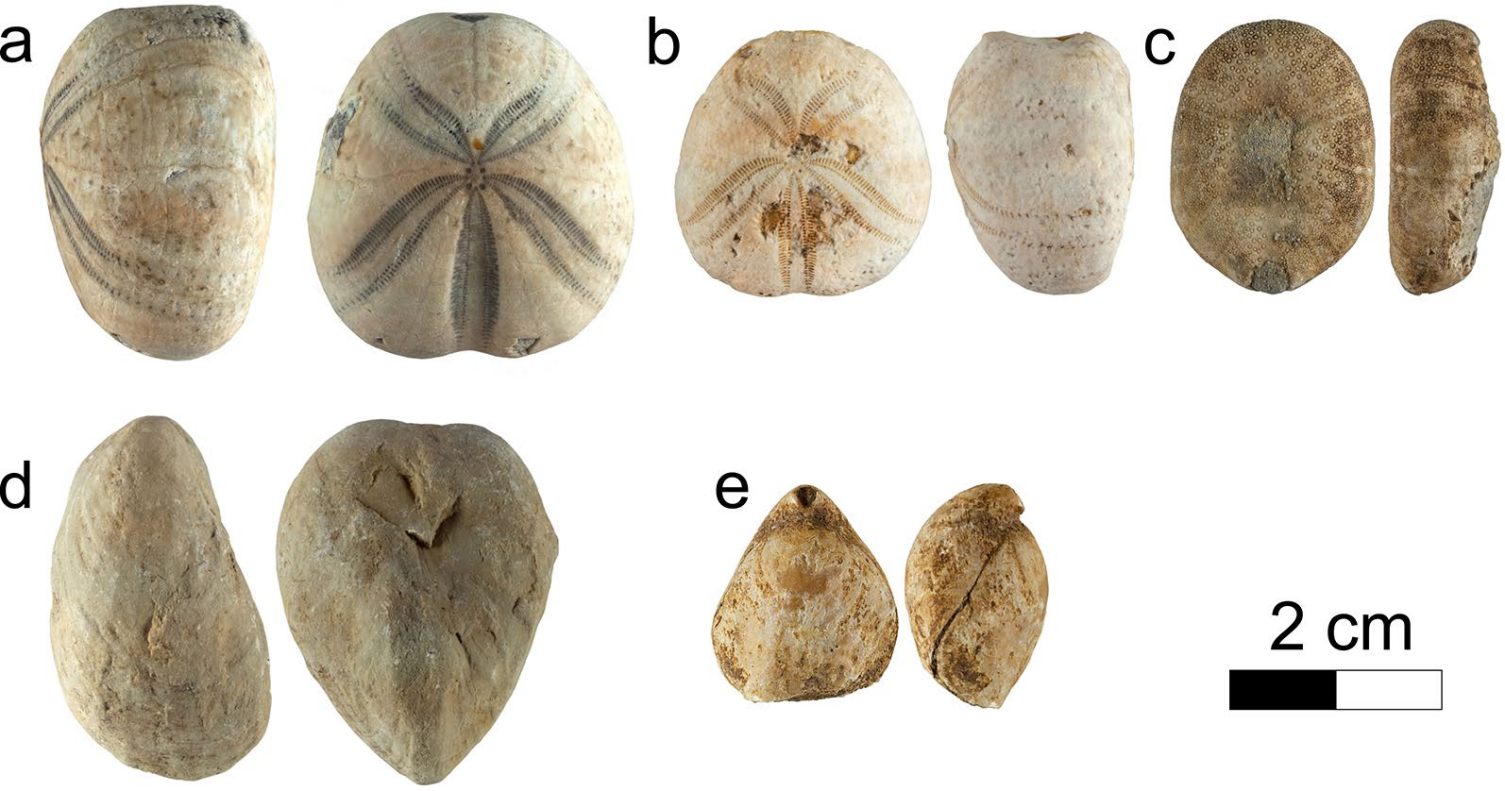
Fossils from the Rivière Member, lower upper to middle upper Barremian: **a** *Heteraster couloni* Agassiz 1839, globose morphotype, La Rivière, Ain, France, MGL.107310 coll. A. Pictet. **b** *Heteraster couloni* Agassiz 1839, globose morphotype, Croy, Vaud, Switzerland, MGL.107311 coll. A. Pictet. **c** *Pygaulus desmoulinsi* Agassiz 1847, La Rivière, Ain, France, MGL.107312 coll. A. Pictet. **d** *Pholadomya* sp., La Rivière, Ain, France, MGL.107313 coll. A. Pictet. **e** *Sellithyris sella* (Sowerby 1823), La Rivière, Ain, France, MGL.107314 coll. A. Pictet

The member, which usually overlays the Fort de l'Écluse Member, is defined by an alternation of grey marls or fine-grained, pyrite-rich, limestones, and white rudist-rich [*Requienia* sp. and *Agriopleura marticensis* (d’Orbigny 1850)] or coarse-grained limestone units. The marly intercalations are especially rich in the echinoid *Heteraster couloni* (Agassiz 1839, Fig. 18a, b) associated to rare *Pygaulus desmoulinsi* Agassiz 1847 (Fig. 18c), *Astrolampas productus* (Agassiz 1836), *Pseudocidaris clunifera* (Agassiz 1840), brachiopods such as *Sellithyris sella* (Sowerby 1823, Fig. 18e) and *Loriolithyris russillensis* (de Loriol 1864), and gastropods such as *Natica (Amauropsis) bulimoides* (Deshayes in Leymerie 1842). Microfossils such as ostracods are also very abundant in these marls. A rich ostracod association was collected near Farges (Ain, France) by Sauvagnat et al. (2001) in the Rivière Member with species as *Bairdia* sp., *Bairdia* aff. *acuminata* Wilkinson 1988, *Dolocytheridea intermedia* Oertli 1958, *Schuleridea* sp., *Centrocythere* cf. *bordeti* Damotte & Grosdidier 1963, *Strigosocythere ? reticulata* Sauvagnat 1999, and *Cythereis (R.) glabrella *sensu Sauvagnat 1999. Each carbonate bundles are topped by an encrusted hardground, while the last marly level grades upward to the Vallorbe Member. The muddy and confined facies of the Rivière Member are considered by Conrad and Ducloz (1977, p. 132) as deposited in upper deep circalittoral environments following a transgressive episode.

#### Subdivisions, geographic distribution and lateral variations

The member is mostly restricted to the Meridional Jura between, Bellegarde-sur-Valserine to the southwest, to the Salève mountain to the southeast, and La Cure to the northeast (Conrad & Ducloz, 1977). The muddy and confined facies of the Rivière Member progressively disappear in direction of the Central Jura, replaced by the outer-platform, coral-rich and oxygenated facies of the Bôle Member (Schardt, 1892). Occasional eastward occurrence of the Rivière type-facies can be observed inside the Bôle Member like along the road 9, 1 km south from Croy (Coordinates 2526′893/1171′235 system CH1903/LV03), intercalated between the GDO Fm and the Vallorbe Member.

#### Biostratigraphic data and age

The Rivière Member was initially reported to the middle to late Barremian by Conrad (1969) and then lowered in the lower *T. Hugii* Zone on the basis of sequence stratigraphic interpretations and orbitolinids dating (Charollais et al., 2013b). Although occasionally present in the latest-most Hauterivian, the numerous *Heteraster couloni* (Agassiz 1839) observed in this member present typical Barremian globose morphotypes (Fig. 18a, b). The echinoid *Pygaulus desmoulinsi* Agassiz 1847 (Fig. 18c), present in the uppermost marly intercalation is restricted to the middle late Barremian–early Aptian time interval. The Barremian rudist *Agriopleura marticensis* (d’Orbigny 1850) indicates an age not younger than the lower *G. sartousiana* Zone (Masse & Fenerci-Masse, 2011). By their identical stratigraphical position and faunal association with the Bôle Member, the Rivière marls can be reported to the *K. compressissima* to the early *G. sartousiana* ammonite zones. The late early Barremian series of the Rivière Member are often missing because eroded or not deposited, as indicated by the dominant late Barremian fauna.

### Vallorbe Member

The Vallorbe lithostratigraphic unit was introduced by Strasser et al. (2016) to replace old informal terms such as ‘‘Urgonien supérieur’’, ‘‘Urgonien blanc’’ (Custer, 1928; Desor & Gressly, 1859), or “Membre[s] des Calcaires urgoniens” (Conrad, 1968, 1969) (Fig. 3). It should be noted that the “Couches du Mormont/Mauremont” from de Tribolet (1856, 1857, 1859) were historically not retained since this ambiguous name was proposed at the same time by two authors for two different formations (see Fig. 3) and subject to controversies between the two antagonists. Strasser et al. (2016) considered the “Urgonien blanc” facies as relatively isochronous throughout the Jura Mountains and so chose the Vallorbe outcrop as type section for its fully visible and complete sedimentary succession, an undeniable advantage compared to the incomplete “Couches du Mormont/Mauremont” from de Tribolet. New field observations and dating allow to consider the Bôle and Rivières members as an isochronous platform drowning succession. This has the consequence that the “Vallorbe Formation” thus only represents the uppermost limestone unit of the “Urgonian blanc” series and is mostly restricted to the platform border in the Central Jura. Consequently, we decided to retrograde the “Vallorbe Formation” of Strasser et al. to a member rank. Moreover, the “Marnes de la Russille” sensu Schardt and Dubois (1902) (i.e. their topmost layer taken as the base of the “Vallorbe Formation” in its initial definition) have to be removed from this unit and incorporated into the emended Gorges de l’Orbe Formation, as discussed above (Fig. 3).

#### Type locality

**Fig. 19:**
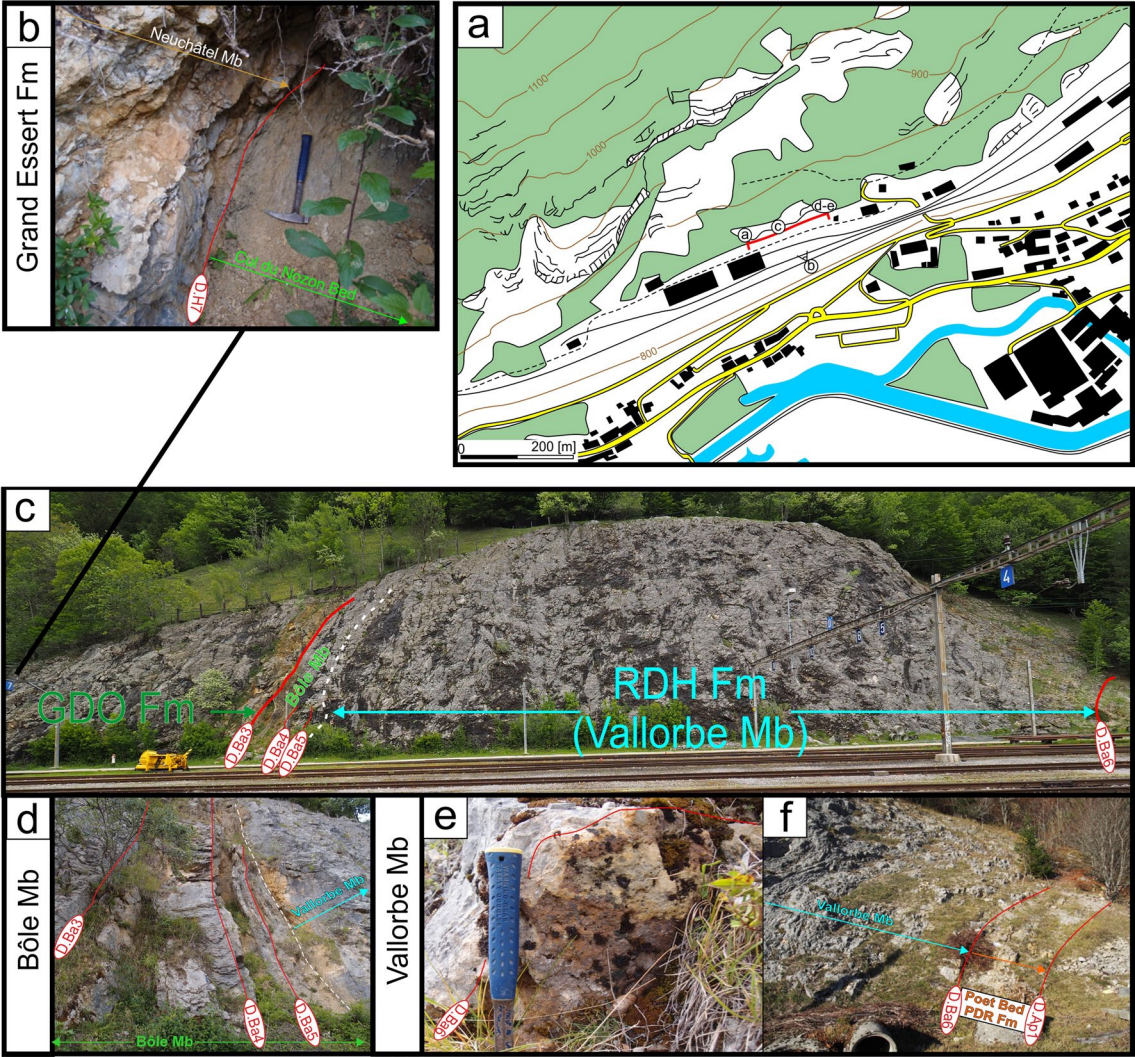
**a** Location map of the type-sections of the Vallorbe Member along the western side of the Vallorbe train station (Vaud, Switzerland) and field-photographies. **b** Boundary between the Neuchâtel Member (GE Fm) and the Cul du Nozon Bed (GDO Fm). The Pont des Pierres Bed is missing here, leaving a lot of glauconite grains on top of the discontinuity surface D.Ha7. **c** General view of the Vallorbe section. The lower limestone unit present transitional facies between the Fort de l’Écluse Member (RDH Fm) and the La Vaux Bed (GDO Fm). **d** View of the Bôle Member and its three main marly intercalations above discontinuity surfaces D.Ba3, D.Ba4 and D.Ba5. **e** Focus on the strongly ferruginous and karstified surface D.Ba6. **f** Upper boundary of the Vallorbe Member with the Poet Bed (PDR Fm), separated by the discontinuity surface D.Ba6

**Fig. 20:**
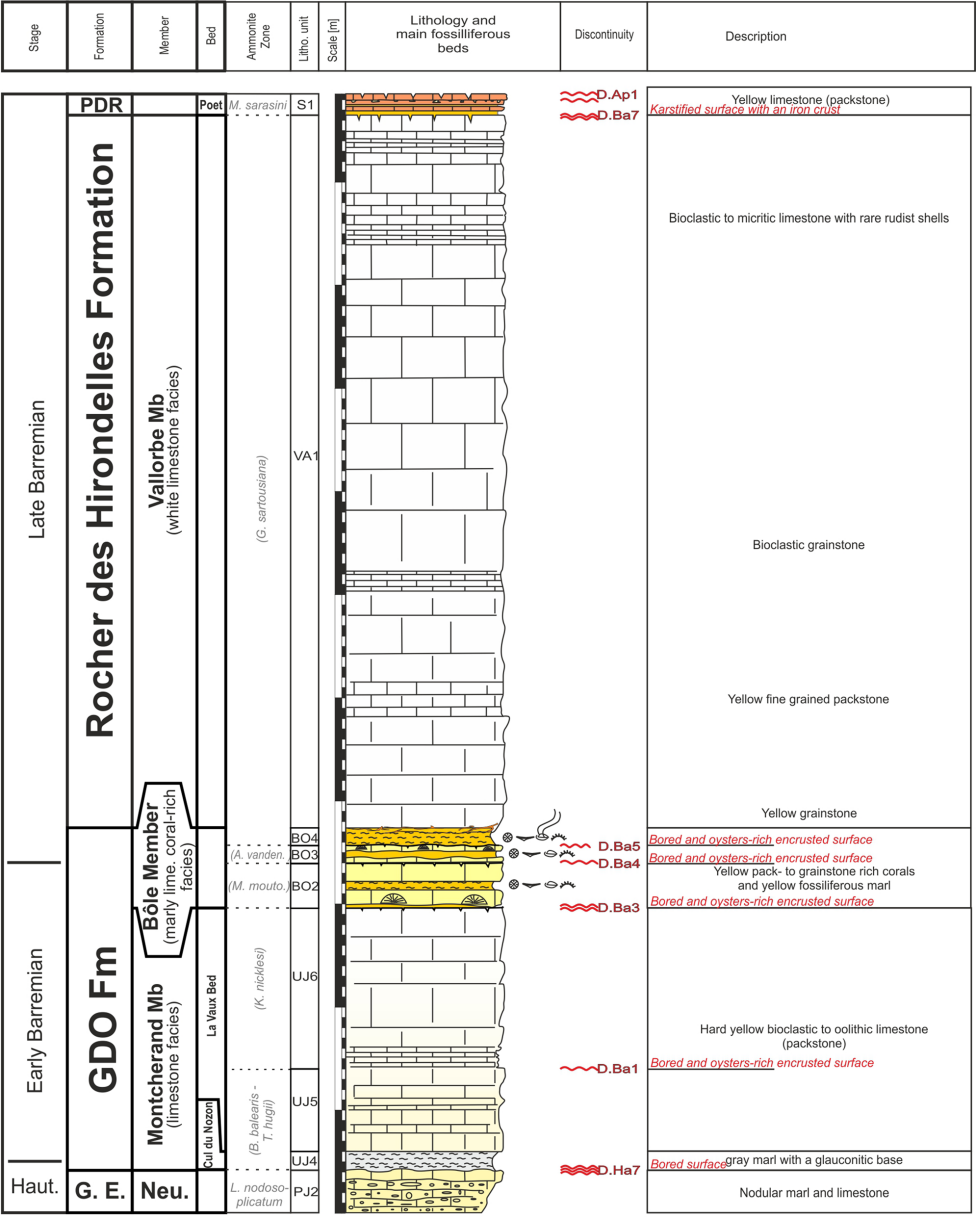
The Vallorbe Member type-section with age, litho- and biostratigraphy on the left side, and the main fossiliferous beds, discontinuity surfaces and description of the log on the right side

As described by Strasser et. al. (2016), the type locality is a steep cliff located close to the Vallorbe railway station (Fig. 19; Coordinates 2517′956/1173′922 system CH1903/LV03). The layers are vertically tilted and are intersected perpendicularly by the railways. The section (Fig. 20) was previously studied by Nolthénius (1921), Conrad and Masse (1989) and Blanc-Alétru (1995) who gave a detailed sedimentological description.

#### Thickness

The Vallorbe Member presents its maximum thickness in the Montricher-Gorges de l’Orbe-Vallorbe area (Jaccard, 1869). At the type locality, a thickness of 67 m was measured from a bioturbated surface at the base of the member to the terminal hardground, topped by the yellowish clay at the base of Perte-du-Rhône Formation. Laterally, the member pinches up to few metres toward the inner platform setting (Bellegarde-sur-Valserine area) and disappears toward the outer platform setting (Morteau and Combe-Le Landron areas), replaced by the “Calcaire jaune de Morteau” (Jaccard, 1870).

#### Definition and boundaries

**Fig. 21:**
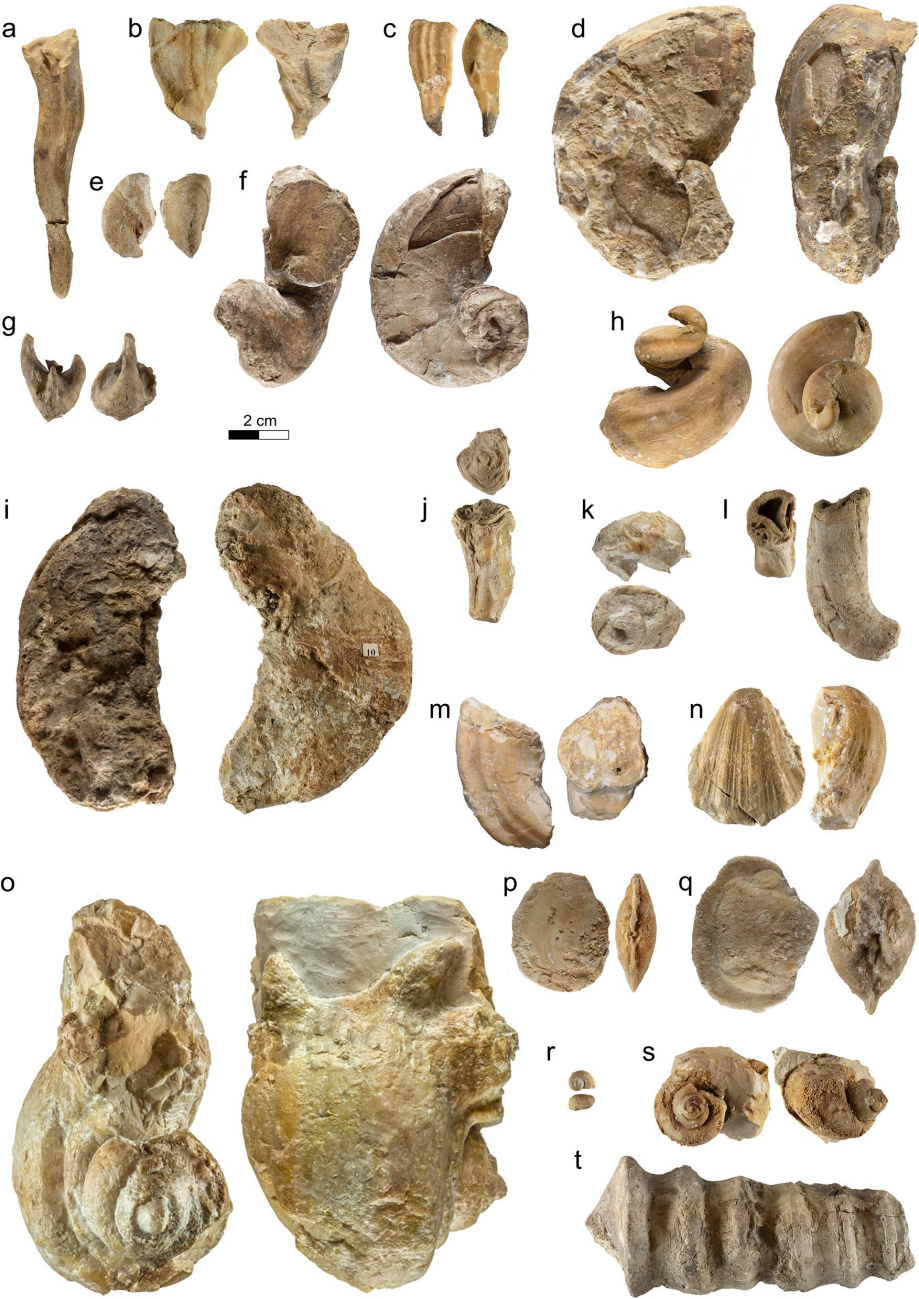
Fossils from the Vallorbe Member, middle upper Barremian: **a** *Debrunia multicarinata* (Matheron 1878), Pompaples plateau, Vaud, Switzerland, MGL.21444 coll. GEOL_REG. **b** *Agriopleura marticensis* ? (d’Orbigny 1850), Montcherand, Vaud, Switzerland, MGL.21445 coll. GEOL_REG. **c** *Agriopleura blumenbachi* (Studer, 1834), Pompaples plateau, Vaud, Switzerland, MGL.107315 coll. Paris GEOL_REG. **d** *Toucasia* gr. *carinata* (Matheron 1842) *lonsdalei* (Sowerby 1837), Perte du Rhône, Ain, France, MGL.107316 coll. GEOL_REG. **e** *Matheronia* sp., Pompaples plateau, Vaud, Switzerland, MGL.21432 coll. GEOL_REG. **f** *Requienia renevieri* Paquier 1903, La Raisse, Vaud, Switzerland, MGL.21410 coll. GEOL_REG. **g** *Monopleura michaillensis* (Pictet & Campiche 1868), Orbe, Vaud, Switzerland, MGL.21463 coll. GEOL_REG. **h** *Requienia ammonia* (Goldfuss 1838), Mormont, Vaud, Switzerland, MGL.21630 coll. GEOL_REG. **i** *Matheronia virginiae* (Gras), L’Auberson, Vaud, Switzerland, MGL.107317 coll. GEOL_REG. **j** *Agriopleura blumenbachi* (Studer, 1834), Pompaples plateau, Vaud, Switzerland, MGL.21425 coll. GEOL_REG. **k** *Requienia pellati* Paquier, Vaulion, Vaud, Switzerland, MGL.21446 coll. GEOL_REG. **l** *Caprinula gracilis*, Pompaples plateau, Vaud, Switzerland, MGL.21435 coll. GEOL_REG. **m** *Debrunia favrei* (Matheron 1878), Mormont, Vaud, Switzerland, MGL.107318 coll. GEOL_REG. **n** *Neithea atava* (Roemer 1839), Mormont, Vaud, Switzerland, MGL.107319 coll. GEOL_REG. **o** *Harpagodes pelagi* (Brongniart 1821), Carrière des Buis near La Sarraz, Vaud, Switzerland, MGL.107320 coll. GEOL_REG. **p** *Sucina germani*, Pompaples plateau, Vaud, Switzerland, MGL.107321 coll. GEOL_REG. **q** *Astarte beaumonti* Leymerie 1842, Pompaples plateau, Vaud, Switzerland, MGL.107322 coll. GEOL_REG. **r** *Nerita* sp., Pompaples plateau, Vaud, Switzerland, MGL.21433 coll. GEOL_REG. **s** *Natica bulimoides* d’Orbigny, Pompaples plateau, Vaud, Switzerland, MGL.21430 coll. GEOL_REG. **t** *Nerinea coquandiana* d’Orbigny 1842, La Presta, Neuchâtel, Switzerland, MGL.21416 coll. GEOL_REG

The Vallorbe Member, starting on top of the Bôle or the Rivière Member, is characterized by a white, massive, limestone cliff containing a rich lagoonal fauna characterized by rudist-shells [*Debrunia multicarinata* (Matheron 1878) Fig. 21a, *Agriopleura marticensis ?* (d’Orbigny 1850) Fig. 21b, *Agriopleura blumenbachi* (Studer, 1834) Fig. 21c, j, *Toucasia* gr. *carinata* (Matheron 1842) *lonsdalei* (Sowerby 1837) Fig. 21d, *Matheronia* sp. Figure 21e, *Requienia renevieri* Paquier 1903 Fig. 21f, *Monopleura michaillensis* (Pictet & Campiche 1868) Fig. 21g, *Requienia ammonia* (Goldfuss 1838) Fig. 21h, *Matheronia virginiae* (Gras) Fig. 21i, *Requienia pellati* Paquier Fig. 21k, *Caprinula gracilis* Fig. 21l, *Debrunia favrei* (Matheron 1878) Fig. 21m], other bivalves [*Neithea atava* (Roemer 1839) Fig. 21n, *Sucina germani* Fig. 21p, *Astarte beaumonti* Leymerie 1842 Fig. 21q] and gastropods [*Harpagodes pelagi* (Brongniart 1821) Fig. 21o, *Nerita* sp. Figure 21r, *Natica bulimoides* d’Orbigny Fig. 21s, *Nerinea coquandiana* d’Orbigny 1842 Fig. 21t]. The Vallorbe Member ends in all sections by a strongly karstified surface (Bertschy, 1958; Charollais et al., 1994; Godet et al., 2016) of variable age, itself often overcome by the Poet Bed of the Perte du Rhône Formation (see Pictet et al., 2016a, 2019).

#### Subdivisions, geographic distribution and lateral variations

The Vallorbe Member includes 2 subunits at its top: (i) the Bellegarde Bed in the Meridional Jura; (ii) the Serrières Bed in the eastern Central Jura.

**Fig. 22:**
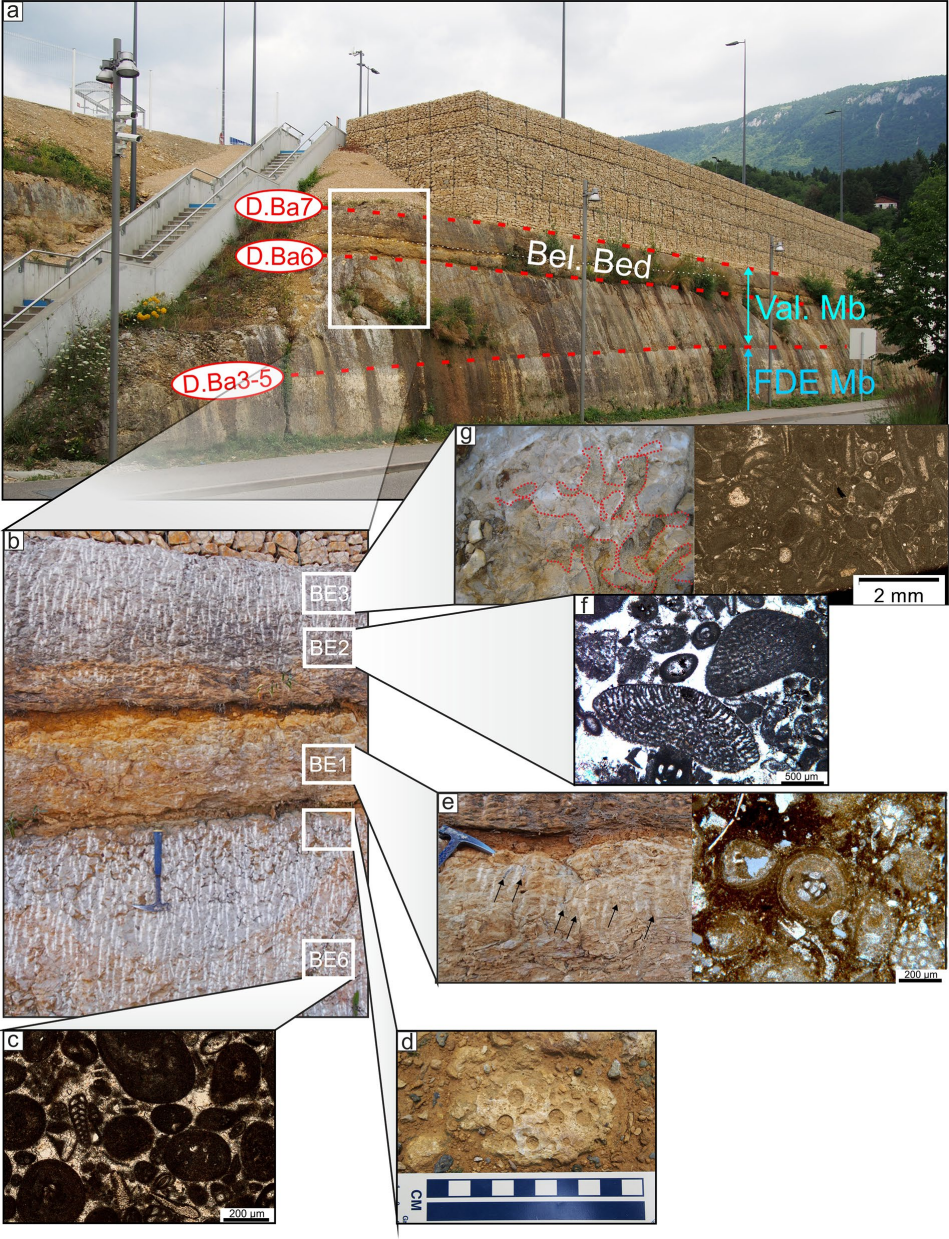
**a** The Bellegarde-sur-Valserine train station outcrop showing the staked Fort de l’Écluse Member, the Vallorbe Member and its Bellegarde Bed in their type-section. **b** Focus on the Bellegarde Bed and its brackish to lacustrine brown marls and limestones topped by a last rudists-rich carbonate bed. **c** Thin section of sample BE6 showing a foraminifera-rich grainstone. **d** Focus on the strongly bored discontinuity surface D.Ba6. **e** Focus on the brown brackish to lacustrine sediments penetrated by numerous rhizoliths, and view of a thin section in sample BE1 showing an oncoidal-rich altered packstone with ostracods and charophytes. **f** Thin section in sample BE2, from the urgonian limestone above, characterized by a foraminifera-rich grainstone. **g** Focus on the karstic pockets on top of this last urgonian bed, filled with sediments of the above Poet Bed (PDR Fm). As visible in thin section of sample BE3, the top of urgonian bed itself is characterized by a strongly micritised bioclastic packstone

The Bellegarde Bed is described in Pictet et. al. (2019), at the top of a cliff located in front of the Bellegarde-sur-Valserine railway station (Fig. 22; Coordinates 5.82564 m E/46.11122 m N system WGS84, projection UTM, zone 31 T). This Bellegarde Bed, which does not exceed 1.5 m thickness, overlay the main sedimentary body of the Vallorbe Member by the intermediary of a karstified surface (discontinuity surface D. Ba6, D4 of Pictet et. al., 2019) with corroded rudist shells infilled by *terra rossa*. This discontinuity surface is topped with yellow to orange brackish/freshwater marls with reworked lacustrine limestone pebbles and well-developed root marks. The marls yielded numerous microfossils including benthic foraminifera, marine or brackish/lacustrine ostracods, dasycladacean algae and charophytes listed in Pictet et. al. (2019, p. 634). These marls progressively pass to a rudist-bearing limestone bed (Unit U2 in Pictet et al., 2019), itself karstified to its top and filled with bioclastic brachiopod-rich sediments of the Poet Bed (Perte-du-Rhône Formation). In our current knowledge, the Bellegarde Bed is restricted to the Bellegarde-sur-Vaserine palaeovalley.

**Fig. 23:**
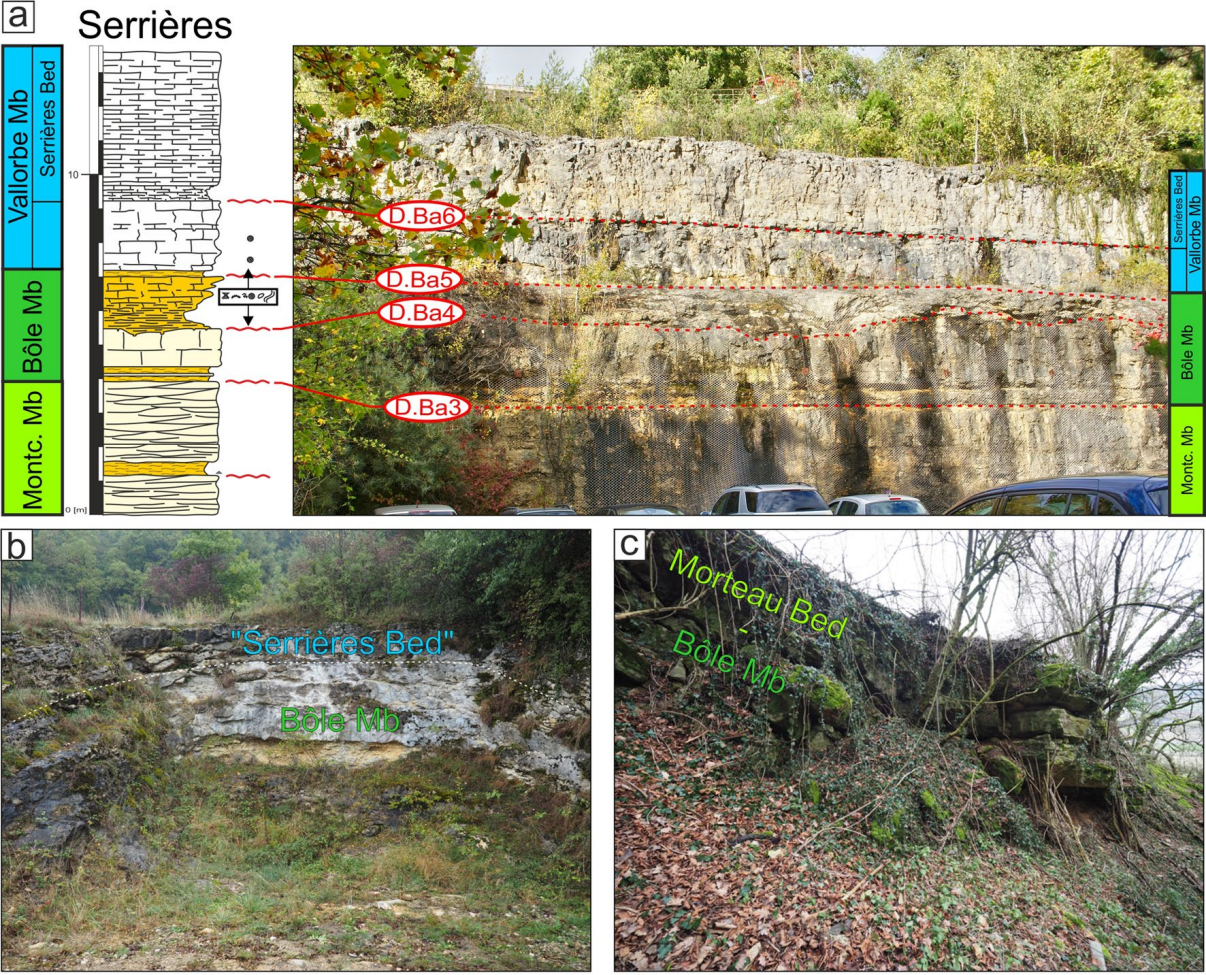
**a** The old Serrières quarry in front of the Philip Morris building, in the western side of the Neuchâtel city. The Vallorbe Formation is characterized here by two white limestone beds separated by the karstic surface D.Ba6. The upper bed is presently named Serrière Bed. **b** Old quarry at the western side of Cornaux (Neuchâtel canton) showing the strong reduction in thickness of the Serrières Bed, directly topping the Bôle Member. **c** Marl-limestone alternation of the undifferentiated Morteau Bed and Bôle Member on the Combes section

The Serrières Bed probably constitutes a bioclastic limestone lens positioned on the outer border of the shallow-water Urgonian platform, which could be relatively contemporaneous to the Bellegarde Bed. Its first eastward observed occurrence is situated in the Serrières district of the Neuchâtel city (Fig. 23a; Coordinates 2558.883/1203.459 system CH1903/LV03) where it overlays the main sedimentary body of the Vallorbe Member by the intermediary of a karstified surface (discontinuity surface D. Ba6). Here, the Serrières Bed measures the same thickness as the below main bed of the Vallorbe Mb with around 5 m thick, while in the nearby eastward Le Mail section, the main bed pinches with a thickness of 1.2 m, the Serrières Bed representing the main white limestone body with its 5 m thick. The Serrières Bed equally pinches eastward between Cornaux (Fig. 23b) and Le Landeron area, passing to the Morteau Bed (Fig. 23c).

The Vallorbe Mb is mostly developed in the Central Jura. Lacking in the Bugey area, it reappears and extends to the southwestern anticlines of the Meridional Jura (Annecy—Aix-les-Bains area), before passing to the Lower Urgonian Member of the southern Subalpine Chains. Southward, the formation is present on the Salève mountain, last Jurassian anticline before the northern Subalpine Chains where this limestone unit is represented by the Lower Urgonian Member.

#### Biostratigraphic data and age

As the underlying Bôle and Rivière members are dated at their top to the early *G. sartousiana* Zone (e.g. De Kaenel et al., 2020) and the overlying Poet Bed of the PDR Fm is dated by ammonite to the *M. sarasini* Subzone (Pictet et al., 2019), the Vallorbe Member is reported by framing from the *G. sartousiana* and *I. giraudi* zones. This dating is corroborated by the presence of the gastropod *Harpagodes pelagi* (Brongniart 1821) (e.g. Jaccard, 1893; Thirria, 1836) whose occurrence is situated in the *G. sartousiana* (e.g. Montplaisant Member from the Orgon type locality, Aubert et al., 2012; Masse, 1976) and to the *M. sarasini* zones (*Harpagodes pelagi* Event in Pictet et al., 2019).

The main limestone body can be more precisely dated by the occurrence of the rudist-shell *Agriopleura marticensis* which indicates an age not younger than the lower *G. sartousiana* Zone (Masse & Fenerci-Masse, 2011), allowing to restrict the age to the lower *G. sartousiana* Zone.

The brackish/freshwater marls of the Bellegarde Bed yielded numerous microfossils listed by Mojon in Pictet et al. (2019), including benthic foraminifera, marine or brackish/lacustrine ostracods (Fig. 2), dasycladacean algae and charophytes. This microfauna indicates the latest Barremian, somewhere between the upper *G. sartousiana* and the *M. sarasini* zones.

The Serrières Bed did not deliver an informative fauna. However, its stratigraphical position between the main Vallorbe unit and the Poet Bed point to a latest Barremian age, upper *G. sartousiana*–*I. giraudi* zones.

## Discussion

### Filing sequences and sequence stratigraphic interpretations

**Fig. 24:**
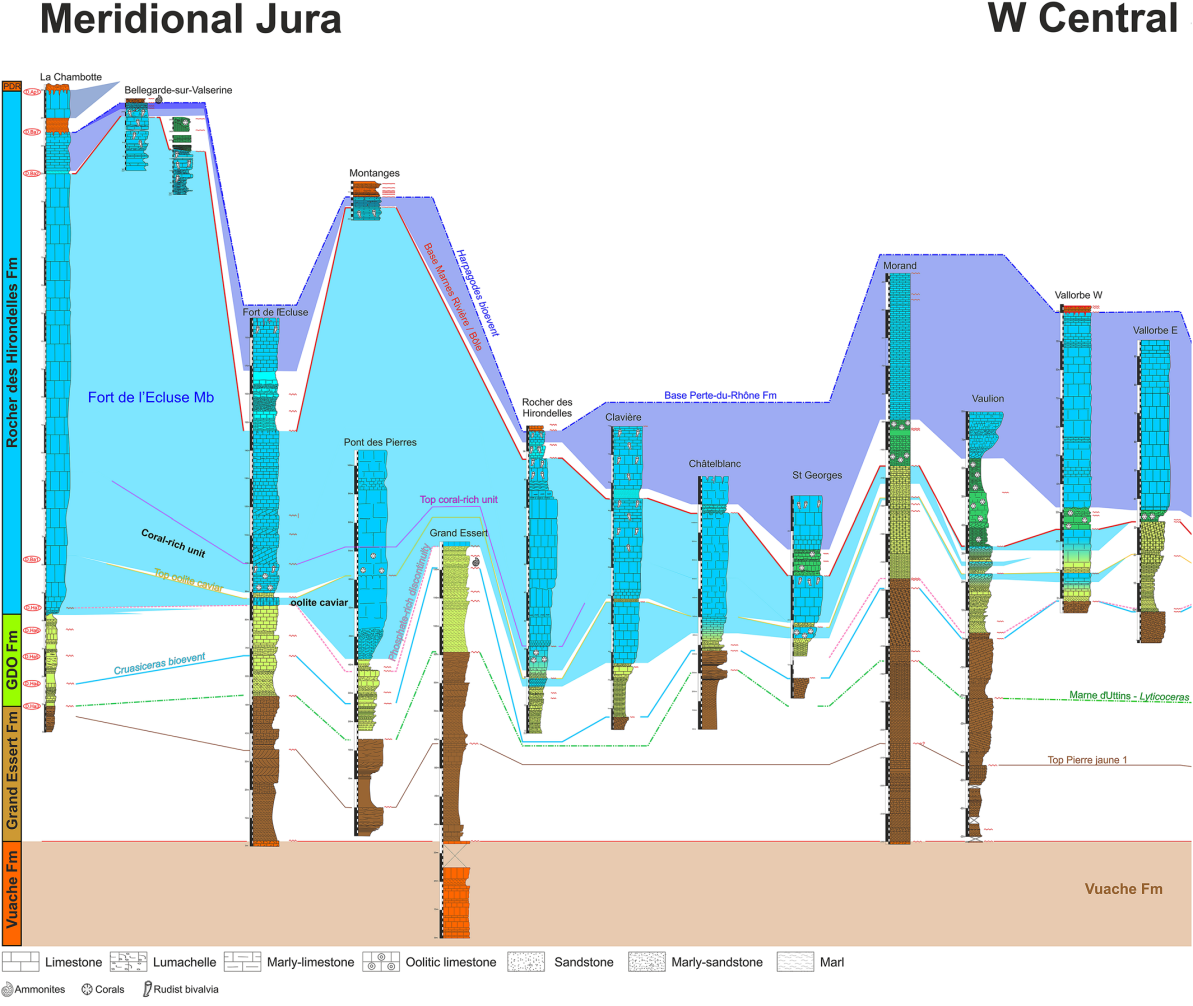

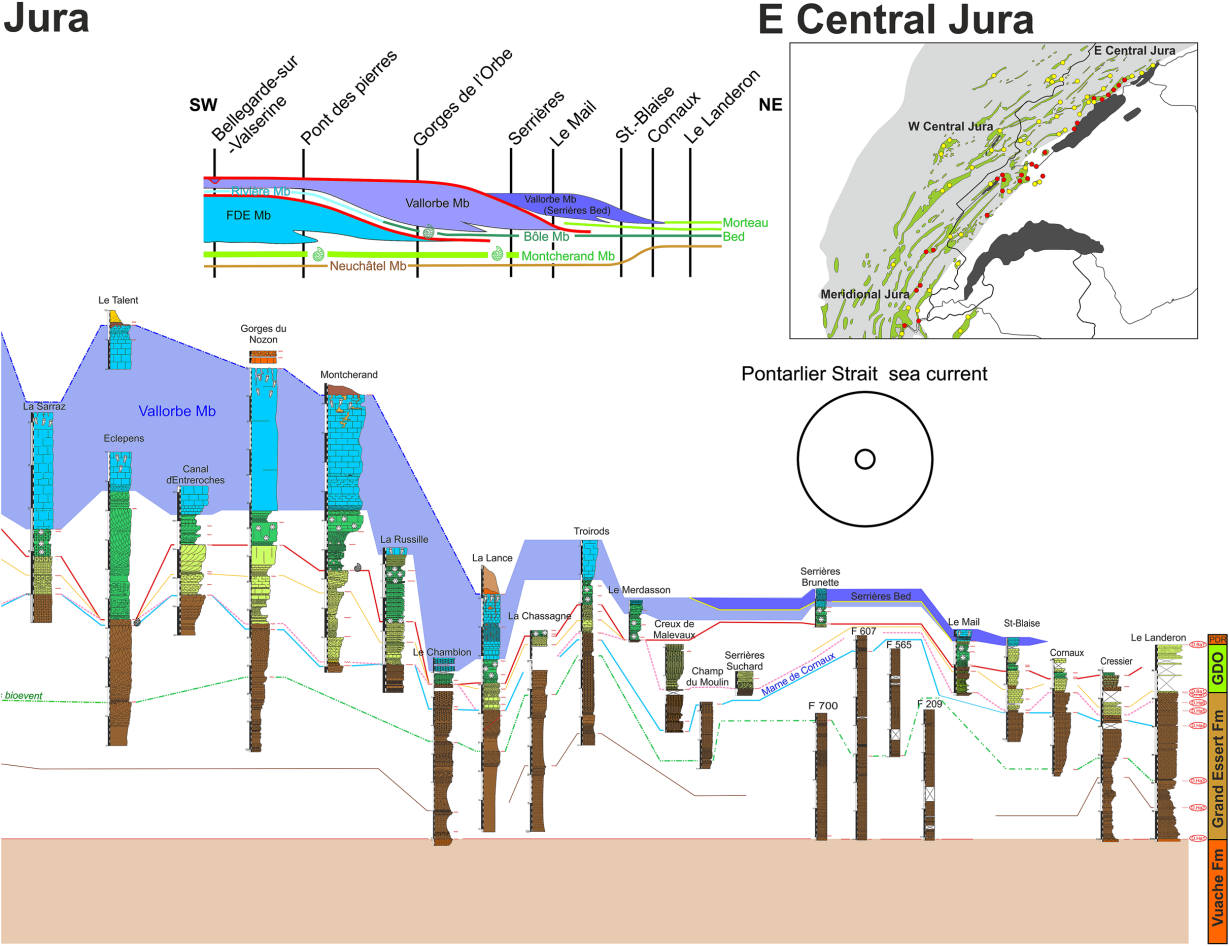
Transect across the Jura platform from the internal to external part following a SW to NE axis along the Jura High chain. Key-events, beds, and surfaces used for correlations between sections are marked by the coloured lines, and the rare ammonite findings are reported on the right side of the logs. Major discontinuities are indicated by thicker lines. This figure highlights the Fort-de l’Écluse carbonate platform (in light blue) followed by the prograding Vallorbe carbonate platform (in purplish), and the Serrières Bed in lowstand prograding wedge (in dark blue). Both urgonian-facies are separated by the flooding series of the Rivière (in turquoise) and Bôle (in dark green) members, and grade north-eastward to the slope deposits of the Gorges de l’Orbe facies

**Fig. 25:**
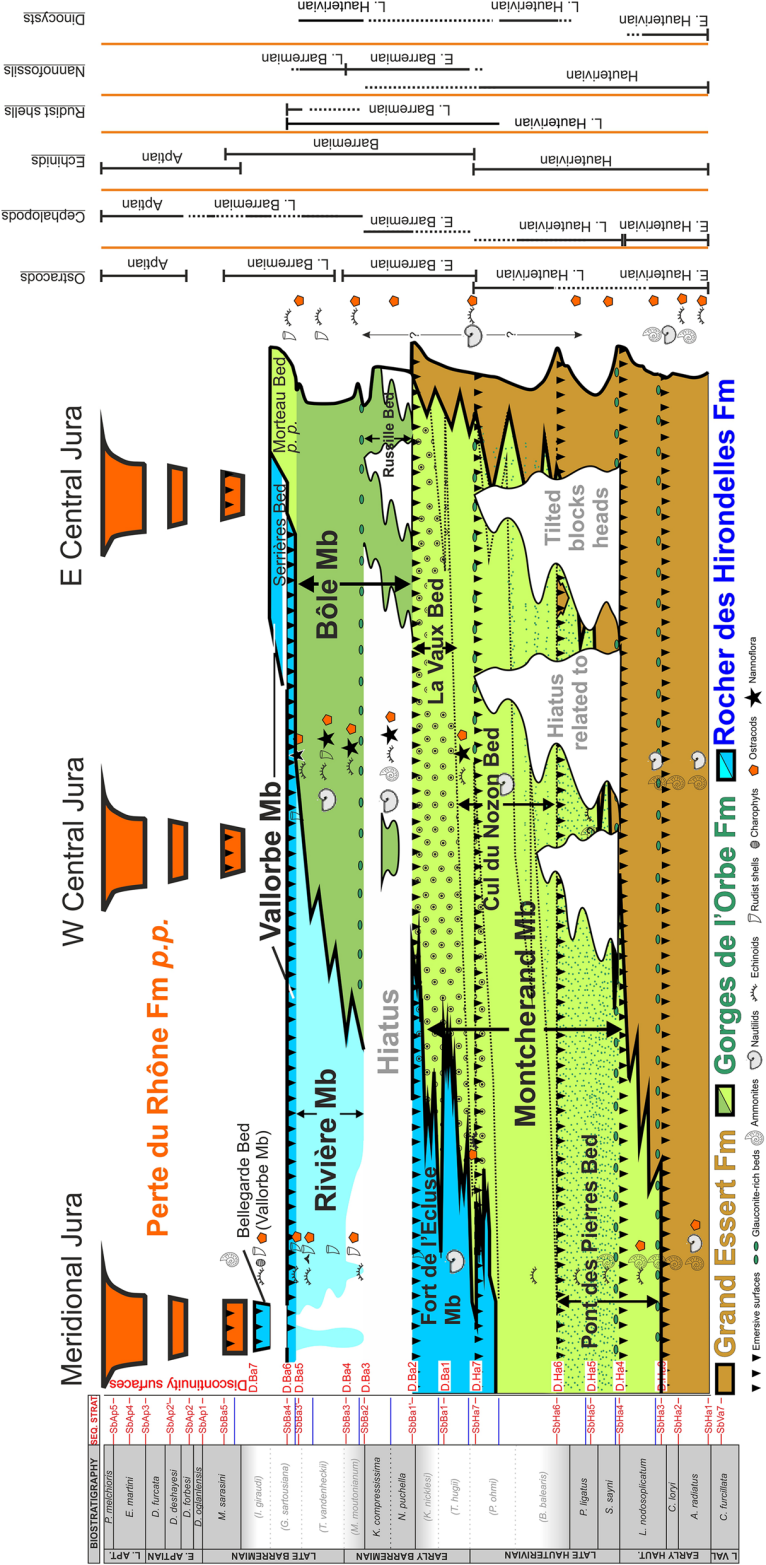
Synthetic scheme of the Hauterivian to Aptian series of the Jura Mountains following a SW to NE axis with the sedimentological units characterizing these. Stages, ammonite biozones and sequence stratigraphic interpretations are reported on the left side, next to main discontinuity surfaces. Grey zones are directly dated by cephalopods, while white biozones are indirectly suggested by sequence stratigraphic interpretations. Main biostratigraphic fossils are reported on the scheme, as well the general dating of these organisms on the right side of the figure

The Jura Mountains provide a unique sedimentary record of the complexity of very shallow-water carbonate platforms developed on a tectonic threshold, which was strongly affected by syntectonic activity, at a time of profound oceanographic and climatic change. Correlations between sections are based on the sedimentary bodies and their vertical and horizontal facies changes, on the sequential stratigraphic interpretations, and on biostratigraphic fossils and bioevents (Figs. 24 and 25). The following sequence-stratigraphic interpretation is based on major lithological changes, on key-beds, and on key-surfaces (hardgrounds, firmgrounds, karstic surfaces, condensed intervals) with: (i) sequence boundaries and associated transgressive surfaces marked by carbonated hardgrounds showing erosive surfaces with karstification, oolite cortex dissolution, borings, large oyster-encrustations, and sometimes iron, glauconite, or phosphate crusts; (ii) maximum flooding surfaces with marly beds showing the deepest facies, often associated with the presence of cephalopods. These sequences stratigraphic nomenclature is formally calibrated only where precise biostratigraphic data are available, mostly in outer platform setting and in marly episodes related to drowning events of the carbonate platform.

The Urgonian carbonate platform was affected by a succession of drowning events which were recorded as deeply marked timelines in the sedimentary succession. Twelve drowning events (D.Ha3 to D.Ba7) were recorded, seven of them being considered as major events (D.Ha3-4-6-7 and D.Ba2-3-6). Some of them are dated by ammonites, which reinforce a chronological framework in which the regional evolution of environments of deposition can be interpreted.

D.Ha3 discontinuity surface is a well-visible hardground on top of carbonates of the Meridional and western Central Jura, which truncates the underlying cross-stratified so-called “Lower Pierre jaune de Neuchâtel”, exhibiting an increase in glauconite and iron oxide contents as well as various bioturbations and borings including *Gastrochaenolites* (Leymerie 1842) or other trace fossils (Godet et al., 2016). D.Ha3 is associated to a drowning event characterized by the wide distribution of the ammonite genus *Lyticoceras* in the Marnes des Uttins (Busnardo & Thieuloy, 1989; Charollais et al., 1989; Clavel & Charollais, 1989a; Godet, 2006; Godet et al., 2010; Strasser et al., 2018). The sedimentation associated to this drowning is strongly enriched in glauconite and phosphates (Godet et al., 2010).

D.Ha4 discontinuity surface is an erosive and perforated boundary associated to a sharp surface and to a marked sedimentary change toward more glauconitic sediments (Godet et al., 2010, 2016). The surface is encrusted by oysters, mineralized with dark green glauconitic grains, and heavily bored by Skolithos-like perforations. The underlying cross-stratified Pierre jaune de Neuchâtel facies is often truncated and channel structures are filled by a mud-supported, calcareous conglomerate, with clasts measuring up to 20 cm (Godet et al., 2010, 2016). Its ammonite dating corresponds to a major sea-level fall which is documented in the upper part of the *Lyticoceras nodosoplicatum* Zone (Hardenbol et al., 1998; Hoedemaeker, 1995a; Sahagian et al., 1996), followed by a rapid transgression during the earliest late Hauterivian *Subsaynella sayni* Zone (Van de Schootbrugge et al., 2003). This eustatic event is associated to the wide dispersion of the ammonite genus *Cruasiceras* like in the Pont des Pierres Bed of the Meridional Jura and in the highly condensed interval making the transition between the Pierre jaune de Neuchâtel and the Pont des Pierres facies in the Central Jura.

D.Ha6 discontinuity surface is marked by the widespread deposit of the lower part of marly Cul du Nozon Bed in most of the Meridional Jura Mountains. This event is associated to the echinoids turnover with the replacement of the dominant species *Toxaster retusus* by the stepped apparition of *Heteraster couloni* and *Toxaster seynensis* during the late Hauterivian *B. balearis* Zone (Clavel in Charollais et al., 2009, Clavel et al., 2014).

D.Ha7 discontinuity surface is a well-marked hardground associated to abundant glauconite and phosphate encrustations. This surface is associated to the widespread deposit of the upper part of the marly Cul du Nozon Bed in most of the Jura Mountains, characterized in the Meridional Jura by sandy marls, dolomite, ferruginous grainstones and several rhizolith-rich surfaces, which form a benchmark level throughout the Meridional Jura. The vegetation of the paleosols must certainly correspond to temporary mangrove equivalents, because the absence of lacustrine deposits. This emersive event, also recognized by Carozzi (1953) and Charollais and Lombard (1966), plays a pivotal role in the Urgonian sedimentation with an important development phase of the RDH Fm and its lagoonal white rudistic limestones. This event affecting the Jura Domaine is correlated to the major sea-level fall documented in the uppermost Hauterivian series, upper part of the *P. picteti* Subzone (Company et al., 2005; Hoedemaeker, 1995b).

D.Ba2 discontinuity surface records an important karstification phase which terminates the photozoan-hetorozoan mixed carbonate production on top of the Fort de l’Écluse Formation–Montcherand Member, associated to the formation of palaeovalleys like in Musinens (Bellegarde-sur-Valserine). The subsequent drowning series follow with the Marne de la Russille Bed, recorded in the form of infills of paleovalleys situated along faults like the Vuache, the Eclépens and the Gorges de l’Orbe faults, as for the PDR Fm (Pictet et al., 2019), while the main part of the Jura Mountains is still subject to an emersion phase linked to a large sea-level fall during the late early Barremian. This event is parallelized with the sea-level fall documented in the middle of the early Barremian *K. compressissima* Zone by Hoedemaeker (1995a). This hiatus is also pointed out by the microfossils dating the base of the upper series of the Bôle Member (De Kaenel et al., 2020), which, very often, directly overcome the Montcherand Member. These microfossils indicated the late early Barremian *M. moutonianum* ammonite Zone, pointing to the non-deposition and/or the erosion of the Marne de la Russille Bed. Pictet in Frau et al. (2018) considers the hiatus of the *N. pulchella* Zone on carbonate platforms as a result of a short-term emersion, consistent with the record of Vocontian marginal settings in which this zone is highly condensed (Arnaud, 2005; Delanoy et al., 2012; Vermeulen et al., 2013), lacking (Arnaud, 2005; Delanoy, 1992), or associated to debris-flows and turbidites (Busnardo et al., 2013). When present, the basal Marne de la Russille infill is characterized by strongly condensed sediments, rich in glauconitic coated pebbles and phosphate content, and characterized by the wide distribution and short-lived of the brachiopod *Glosseudesia ebrodunensis* (de Loriol 1864). The very small juvenile ammonite related to the late early to early late Barremian spiny Holcodiscidae collected in this bed as well the nannoflora dating (see De Kaenel et al., 2020) allows to refer this event to the preluds of the Mid-barremian event (Fig. 25, MBE, Coccioni et al., 2003; Pictet, 2017). The Marne de la Russille Bed is followed by the wider deposition of the upper series of the Bôle Member in outer platform settings (Central Jura) and the Rivière Member in inner platform settings (Meridional Jura, Schardt, 1892), together with a remarkable succession of drownings. The most south-westward areas from Bellegarde-sur-Valserine record the non-deposition of the Rivière Member (e.g. Fig. 22). The identical micropaleontological fauna and shared species of the macropaleontological associations, the lateral continuity between the Bôle Member and the Rivière Member (Schardt, 1892), and the identical sequential position demonstrate that the Bôle and Rivières members are two time-equivalent facies of an isochronous platform drowning succession. It results that the “Urgonien blanc” limestone is a diachronous facies represented by two successive carbonate platforms in forced progradation, prograding south-, southest- and north-eastward from the Bugey area (Meridional Jura). The fossil record indicates a late early to early late Barremian age for these transgressive series (see also De Kaenel et al., 2020). Time-equivalent drowning series are under studies in the Languedoc platform with the Vire du Serre de Tourre and Seynes Marls (Pictet in Frau et al., 2018, p. 245), and the Drusberg Member of the eastern Helvetic Alps (Pictet et al., 2018), both delivering numerous late early to early late Barremian ammonites (*N. pulchella* to *S. sartousiana* zones) associated to a strong paleoenvironmental shift (Pictet, 2017).

The last notable discontinuity surface D.Ba6 records an important karstification phase (Bertschy, 1958; Charollais et al., 1994; Godet et al., 2016) followed by the final drowning event of the Jurassian Urgonian platform (Fig. 25; see Pictet et al., 2016a, 2019). The Serrières Bed above on the distal border of the carbonate platform could represent some lowstand deposits, while the Bellegarde Bed and its deepening upward sequence filling in the incised valley of Bellegarde-sur-Valserine is more related to the transgressive system tract. Both urgonian lenses are topped by the Poet Bed without a clear discontinuity surface (Figs. 24 and 25). As the Vallorbe Member is not younger than the lower *G. sartousiana* Zone (Masse & Fenerci-Masse, 2011), the Bellegarde and the Serrières beds being reported from the upper *G. sartousiana*–*I. giraudi* zones, and the Poet Bed above having delivered a *Martelites* sp. juv., the duration of the emersion phase can be estimated. In most of the sections, the karstification phase encompasses the upper *G. sartousiana* Zone to the *I. giraudi* Zone included, as recorded on other urgonian carbonate platforms (e.g. Frau et al., 2018; Masse & Fenerci-Masse, 2013). Such dating is consistent with the record of Vocontian outer-ramp settings in which this time-interval is highly condensed (Arnaud, 2005; Masse & Fenerci-Masse, 2013), or associated to debris-flows and turbidites (Busnardo et al., 2013). This emersion phase coincides with the last appearance datum of the rudist genus *Agriopleura* (Masse & Fenerci-Masse, 2011, 2013). An ammonite faunal turnover occurs at that time, especially in the *I. giraudi* Zone, with the last appearance datum of the Pulchelliidae and Hemihoplitinae, replaced by the strong diversification of the Heteroceratidae (Bert et al., 2008). Only the Vuache fault (see Fig. 14), close to Bellegarde-sur-Valserine, had a sufficient subsidence to allow the deposition and/or the preservation of the Bellegarde Bed. The maximum flooding surface coincides with the deposition of the Poet Bed which dates from the *M. sarasini* Subzone (Fig. 25, Pictet et al., 2019).

These key surfaces and associated events that affected the Jurassian Urgonian platform allow to propose a solid correlation of the sections across the mountain range. The Jurassian Urgonian platform is characterized by a succession of mixed photozoan–heterozoan carbonate facies prograding from the Bugey to the Central Jura and to the Alps, with protected outer platform sponges and bivalves-rich marls and limestones (GDO Fm) and winnowed oolitic limestones (Neuchâtel Member *p.p.*) grading to inner platform rudistic limestones (RDH Fm, Fig. 25). The resulting strong diachronous age of the superposed facies prevents any age correlation based on the facies.

### The age controversy of the Jurassian Urgonian platform

The age of the Jurassian Urgonian platform (RDH Fm) has been subject to controversy for more than three decades for which two main chronostratigraphic models faced in the literature. On the one hand, Clavel et al. (2007), Conrad et al. (2011), Charollais et al., (2013a, 2013b) and Strasser et al., (2016, 2018) assigned a late Hauterivian age to a large part of the Urgonian deposits, based on: (i) their biostratigraphic scale of orbitolinids; (ii) nannofossils; (iii) sequence-stratigraphic interpretation; (iv) reinterpretations of strontium-isotope and K–Ar ages from Godet et al. (2011). This age interpretation was refuted by Arnaud et al. (1998), Huck et al., (2011, 2013), Adatte et al. (2005), Godet (2006), and Godet et al., (2010, 2011, 2012, 2013), who inferred a late Barremian age for the “Urgonian blanc” deposits in the Jura Mountains. These authors based their model on: (i) another biostratigraphic scale of orbitolinids; (ii) nannofossils; (iii) sequence-stratigraphic interpretation; (iv) the hypothetic presence of an important hiatus of the uppermost Hauterivian and lower Barremian series based on the interpretation of the numerous limestone “pebbles” present at the base of the GDO Fm as pebbles coming from important reworking and stratigraphic hiati; (v) strontium-isotope and K–Ar ages. This objective study takes into account the observations of both protagonists and proposes a new sedimentological model bringing in these two opposite models in relative agreement with a mostly late Hauterivian to early Barremian age for the RDH Fm in the Geneva region and a middle late Barremian age in the Central Jura. The multiple biomarkers taken into account such as cephalopods, echinoids, rudist shells, ostracods, charophytes, dinocysts and nannoflora allowed a better age consensus and participated to correlate main discontinuity surfaces, as well sedimentological bodies, leading to a radically different sequence-stratigraphic interpretation and an adjoining sedimentary model with an apparent opposite direction of progradation of the Urgonian platform.

Due to the absence of pelagic biomarkers, the Urgonian platforms pose numerous problems from a stratigraphic, palaeontological and paleogeographic point of view. As early as the 1960s, micropaleontolgists tried to solve these problems by studying benthic microfossils such as green algae and orbitolinids. Schroeder et. al. (1968) defined five biozones or successive orbitolinids associations. For these authors, as for others, the Orbitolinidae play an essential role as biostratigraphic markers because the latter show evolutionary trends relating to the size, the external shape and the internal structure of the test, in particular the morphological complexification of the embryo of the megalospheric forms (Schroeder et al., 2002). Subsequently, Clavel et al. (1995), Charollais et al. (1998), or also Granier et al. (2013), attempted to calibrate the extensions of orbitolinids species on the extension of ammonites, on platform edge sections, where ammonite-rich marls and Urgonian limestones meet and intermingle. Over the years, two discordant biozonal scales of orbitolinids appeared, leading to strong age controversies (e.g. Arnaud-Vanneau et al., 2005; Clavel et al., 2007, 2010). In addition to these distensions, recent palaeoecological studies highlighted that some morphological factors such as the shape of orbitolinids are related to paleoenvironmental conditions such as the depth, the water turbidity, etc. Several studies have been published concerning the relationship of orbitolinid test shape (conical, discoidal or flattened) to sea-level changes and sequence stratigraphy (Simmons et al., 2000). Rahiminejad and Hassani (2016) highlighted the dominance of conical morphologies in the sublittoral zone, whereas the almost discoidal and discoidal forms populated the middle part of the proximal sublittoral zone. The present study tends to question the use of orbitolinids as biomarkers. Indeed, regardless of the biozonal scale chosen, the species considered to be the oldest are met in the Central Jura where the outermost facies of the carbonate platform are present, while the so-called youngest species are met in the innermost facies of the Meridional Jura (e.g. Clavel et al., 2007, Fig. 10). With the present sedimentary model, at the complete opposite of the previous studies, data strongly suggests a facies dependence of orbitolinids with a vertical succession of species over time, correlated to the vertical superposition of facies. The same successive associations of orbitolinids seems to occur in a succession of non-contemporaneous sedimentary bodies. Charollais and Lombard (1966, p. 64) expressed the fact that if the Orbitolinids are too closely linked to the facies, it goes without saying that the appearance of these no longer corresponds to a determined geological time, but to the appearance of specific ecological conditions whose causal link to time is not obvious.

Some marly levels such as the Cul du Nozon Bed, at the base of the GDO Fm, have been more conducive to the preservation of organic microfossils (dinocysts). The Morand drilling (VD), studied by Jan du Chêne et al. (2016), provided about 30 species with a temporal range comprised between the late Hauterivian and the early Barremian, which is in accordance with the present paper. Associations of dinocysts harvested at the base of the GDO Fm in the Eclépens and La Sarraz quarries (Clavel et al., 2007; Jan du Chêne et al., 2016) are not considered since their exact levels of harvesting in these atypical sections are not localizable on the present section. A study was carried out in 2006 on the nannofossils taken in the Formation of the Gorges de l'Orbe in the quarries of Eclépens and La Sarraz (Godet, 2006, appendix 5). This blind test study was carried out on the same samples by three specialists, leading to controversial conclusions (Clavel et al., 2007; Godet et al., 2010; Jan du Chêne et al., 2016). Despite of the generally poor preservation, many species were identified. Two specialists established a late Hauterivian age for all studied samples, while on the other hand, another specialist placed most samples in the late Barremian and even in the Late Albian. Still today, no explanation can be given for such a radical difference in the determination of the nannoflora and the ages obtained on the same samples. New analyses were performed on other sections without such important reworkings, channelizations and karstification but unfortunately the strong circulation of fluids and the numerous phases of emersion have greatly deteriorated the preservation of nannofossils, preventing any dating. The quarries of Eclépens and La Sarraz were also recently resampled for the nannofossils and restudied by De Kaenel et al. (2020). A mixture of numerous Boreal and Tethyan taxa was found in the dark marly layers infilling channel structures of the Bôle Member. This nannoflora pointed to precise ages ranging from the late early to the middle late Barremian.

Used from long date for biostratigraphic purposes in the Urgonian facies, the rudist shells are also part of the age controversy. Most of them are consistent with our age model with the genera *Agriopleura*, *Lovetchenia*, *Monopleura*, *Pachytraga*, *Requienia* and *Toucasia* in the Fort de l’Écluse Member (Masse et al., 1998), the genera *Agriopleura*, *Monopleura*, *Pachytraga*, *Requienia* and *Toucasia* in the Bôle and Rivière members (e.g. Astre, 1961), and *Agriopleura*, *Caprinula*, *Debrunia*, *Monopleura*, *Requienia* and *Toucasia* in the Vallorbe Member. The *Pachytraga* beds of the Bôle Member (Astre, 1961; Blanc-Alétru, 1995; Masse et al., 1989), were regarded by Masse et. al (1989) as age diagnostic and were reported to the late Hauterivian by comparison with the well dated Provençal equivalents and on orbitolinids dating. The type material coming from the Bôle Member is presently associated to a “middle” Barremian Holcodiscidae and to the nautilus *Eucymatoceras plicatum* (Fitton 1836) clearly allowing to reject this late Hauterivian age assignment for type-specimens of the Jura Mountains. In the same way, *Requienia renevieri* Paquier 1903, present in the Vallorbe Member, is currently considered as a typical late Hauterivian marker of the Provence platform (Masse et al., 1998). Recent field work as well the literature on the Ardèche and the Helvetic Alps (see Bollinger, 1988; Scholz, 1984; Stössel in Kürsteiner and Klug 2018) show that this rudist species is present in late Barremian Urgonian series and cannot be used as a biomarker. Considering the latest advances, the environmental impact on the geographical distribution of rudists should be more strongly considered. Following Masse and Fenerci-Masse (2008), biostratigraphic distribution of some genera as *Pachytraga* has mainly a regional significance, showing a good correspondence between the regional disappearance of taxa and climatic events controlling the geographic distribution of more sensitive rudists genus.

The use of non-biological tools such as geochemistry, a priori less susceptible to environmental hazards, were used in order to date and correlate the Urgonian series of the Jura Mountains (e.g. Charollais et al., 2013a; Godet et al., 2006, 2011; Granier et al. 2016). The use of such tools is also highly questionable on a shallow-water tectonic threshold affected by numerous emersion phases, freshwater incomings and strong continental influx. Despite this, the use of the C-isotope record as an indirect dating tool has been recently generalized in the Urgonian platform setting (e.g. Bonvallet et al., 2019; Huck et al., 2011, 2013; Stein et al., 2012; Wissler et al., 2003). According to these authors, C-isotope profiles show comparable trends across the platform sections, and therefore, are tentatively used to establish chemostratigraphic correlations with the ammonite-rich basial successions. In recent years, few studies questioned the use of C-isotope chemostratigraphy in the Urgonian platform setting given that the shallow-water carbonate platforms are more subject to a complex and polygenetic diagenetic history (Godet et al., 2016; Huck et al., 2017; Tendil et al., 2019). In fact, platform environments are subject to numerous subaerial exposures, very commons, and sometimes long lasting (Strasser 2015), like the major hiatus recorded in Bellegarde-sur-Valserine, between D.Ba2 and D.Ba7 discontinuity surfaces which covers more than half of the Barremian stage. Such emersive surfaces are associated to non-deposition and erosion phases obliterating large portions of the stable isotopes record (Huck et al., 2017). Subaerial exposures are equally associated to meteoritic diagenesis and to karstification (Godet et al., 2016), but also to continental sedimentation and influxes providing very negative isotopic material contaminating the local signal (Pictet et al., 2019). Between these discontinuity surfaces, commonly developed peritidal cycles in the inner platform environments of the Meridional Jura, typical of the phreatic meteoritic zone (Richard et al., 2007; Volery et al., 2010), which are characterized by a strong covariance between the δ^18^O and δ^13^C signatures (Léonide et al. 2014; Tendil et al., 2019; Volery et al., 2010). The primary diagenetic signal of the Urgonian series was fortunately not altered by the burial diagenesis, which only experienced shallow burial diagenesis during the later Cretaceous (Godet et al., 2016; Moss & Tucker, 1995; Moss, 1992a, 1992b). Nevertheless, the stable isotopes record, as good as it is, as well other chemostratigraphic proxies, is often biostratigraphically poorly constrained or based on unreliable biomarkers, leading to very subjective interpretations (e.g. Charollais et al., 2013a; versus Godet et al., 2011). Application of C-isotope chemostratigraphy in the Urgonian platform environments, therefore, requires a fine understanding of the platform-to-basin diagenetic history to estimate degrees of preservation or of alteration of primary isotopic signatures, which interpretation remains a challenging issue, requiring a great caution (Godet et al., 2016; Huck et al., 2017; Léonide et al., 2014; Tendil et al., 2019).

Finally, the basal conglomerate and its inferred long-time hiatus at the base of the GDO Fm was inspected in detail. The numerous limestone “pebbles” or balls were observed at the base of the GDO Fm and at the base of the Bôle Member respectively. Both conglomerates are mostly represented by autochthonous sponges and intraclasts reworked on the platform slope, in strongly bioturbated levels and in channelized structures like in the Cul du Nozon section. Most of these “conglomerates” are associated with numerous endobiont bivalves made of the same lithological material. The only beds of intense reworking with true pebbles are encountered in the Pont des Pierre Bed from the Central Jura and in the base levels of the Bôle Member in Eclépens Channel. In both lithologies, pebbles are strongly encrusted, bioperforated and occasionally coated with glauconitic and phosphatic crusts (Fig. 7c).

### Progradation of the Urgonian platform

All previous works, rested on two largely accepted historical axioms following which: (i) carbonate platform progradation followed a NE-SW axis based on the strong reduction and disappearance of lithological units in direction of the NE due to a hypothetical proximity of emerged lands (Adatte, 1988; Favre, 1843, 1867; de Loriol & Gilliéron 1869; Steinhauser, 1969; Steinhauser & Charollais, 1971); (ii) main lithostratigraphic units uniformly and contemporaneously deposited through the Jura realm.

New results challenge these historical axioms, suggesting the presence of a reversed depositional model with a carbonate platform/ramp progradation initiating in the present-day Meridional Jura and prograding toward the Central Jura area which was occupied at the time by a marine channel connecting the Tethys Ocean to the Paris Basin (Pictet et al., 2016a, 2019). Sequence stratigraphic interpretations, as well as palaeontological dating indirectly show that during the Berriasian and Valanginian the source of the carbonate production (i.e. Pierre-Châtel and Chambotte formations) was situated in the present-day area of the Bugey in the southernmost Jura chains (Ain, France; Guillaume, 1966; Hennig Fischer, 2003; Hillgärtner, 1998, 1999), and radially prograded toward the Vocontian Basin to the south, and toward the Central Jura to the northeast, with distal outer-platform sediments (i.e. Unité oolithique inférieure, Vions Formation). The Lower Cretaceous sedimentological facies from the Meridional Jura highlight innermost platform palaeoenvironments with strongly developed emersive surfaces (Davaud et al., 1983; Joachimski 1994; Pictet et al., 2019), continental sandstones (Carozzi, 1953; Pictet et al., 2019), charophytes and lacustrine sediments (Joachimski 1994; Mojon, 2002; Pictet et al., 2019), and dinosaur remains (Mojon, 2001) and foot-traces (Charollais et al., 2007; Meyer & Thüring, 2003). Outer platform facies are particularly developed in the Central Jura from the latest early Valanginian onward with the deposition of the ammonite-rich sediments of the Marnes de Villers, Marnes jaunes de Morteau and Marnes bleues d’Hauterive (Baumberger, 1901), Perte-du-Rhône Formation (PDR Fm; Pictet et al., 2016a, 2019), Narlay Formation (Renz et al., 1963). A corollary of these new results is the diachronous age of lithostratigraphic units through the Jura chains during: (i) the Berriasian (diachronism of the facies “Marbre bâtard”, Guillaume, 1966, p. 128); (ii) the Valanginian (Chambotte vs. Vuache formations; Blondel, 1990; Deville, 1990; Hennig Fischer, 2003; Hillgärtner, 1998, 1999); (iii) the Hauterivian (Lower Pierre jaune de Neuchâtel vs. Marnes bleues d’Hauterive and lower part of the “Urgonien jaune” vs. Pierre jaune de Neuchâtel, Pictet et al., 2016b, this study); (iv) the Barremian (“Urgonien blanc” vs. upper part of the “Urgonien jaune”, Pictet et al., 2016b; Schardt, 1892, this study); (v) the Aptian-Cenomanian time interval (across the Perte-du-Rhône Formation and at the boundary with the Narlay Formation, Guillaume, 1966; Pictet et al., 2016a, 2018).

Sedimentological studies more specific to the Urgonian facies in the Meridional Jura highlighted innermost platforms palaeoenvironments characterised by the cyclical development of a freshwater table (Richard et al., 2007; Volery et al., 2010), by the presence of continental sandstones (Carozzi, 1953; Pictet et al., 2019), by charophytes and lacustrine sediments (Mojon, 2002; Pictet et al., 2019) and by the development of macrokarst systems. Major hiatuses are recorded between D.Ba2 and between D.Ba7 discontinuity surfaces which can cover more than half of the Barremian stage. Sedimentological studies of the Urgonian facies in the Central Jura highlighted outer platform environments dominated by sponge-bivalves-bryozoan associations followed by large muddy-coral colonies associated to a very diversified echinoid fauna. Such open-sea environments have been optimal for the study of the nannoflora (De Kaenel et al., 2020), for collecting cephalopods, and for recording a marine isotope signal not altered by early diagenetic processes (Pictet, in prep.). Hiatuses related to emersive episodes disappear northeastward and are replaced by hiatuses related to syntectonic activity. The strong reduction and disappearance of lithological units in direction of the NE is not due to an hypothetical proximity of emerged lands contrary to previous beliefs (Adatte, 1988; Favre, 1843, 1867; de Loriol & Gilliéron 1869; Steinhauser, 1969; Steinhauser & Charollais, 1971) but to the lateral facies changes and to the progressive condensation of the series under the influence of underwater currents moving into the Pontarlier channel, connecting the Tethys ocean to the Paris Basin (Pictet et al. 2016a, 2016b, 2019). Such currents, which were already highlighted by Kiraly (1964, 1965), prevented the development of a carbonate platform in shallow environments.

The present lithostratigraphic study of the Urgonian platform along a SW–NE axis clearly shows a reversed depositional model with a carbonate platform/ramp apparent progradation initiating in the present-day Meridional and prograding toward the Central Jura area (Figs. 24 and 25). The study of sections following other axis also display a progressive progradation especially developed from the external Jura ranges toward the Alps, either from the Jura-Burgundy threshold toward the Tethys ocean (Baumberger, 1901; Hennig Fischer, 2003; Lory, 1857; Pictet et al., 2016a, 2019). Thus, the Urgonian platform seemed to appear in the Bugey area from where it prograded southward to the Vocontian Basin (Charollais et al., 2001, 2003; Clavel & Charollais, 1989b; Clavel et al., 2007, 2014), southest- and eastward to the frontal ditch of the outer crystalline massifs and north-eastward from the Bugey area (Charollais et al., 2009; Détraz et al., 1987).

## Conclusions

**Fig. 26:**
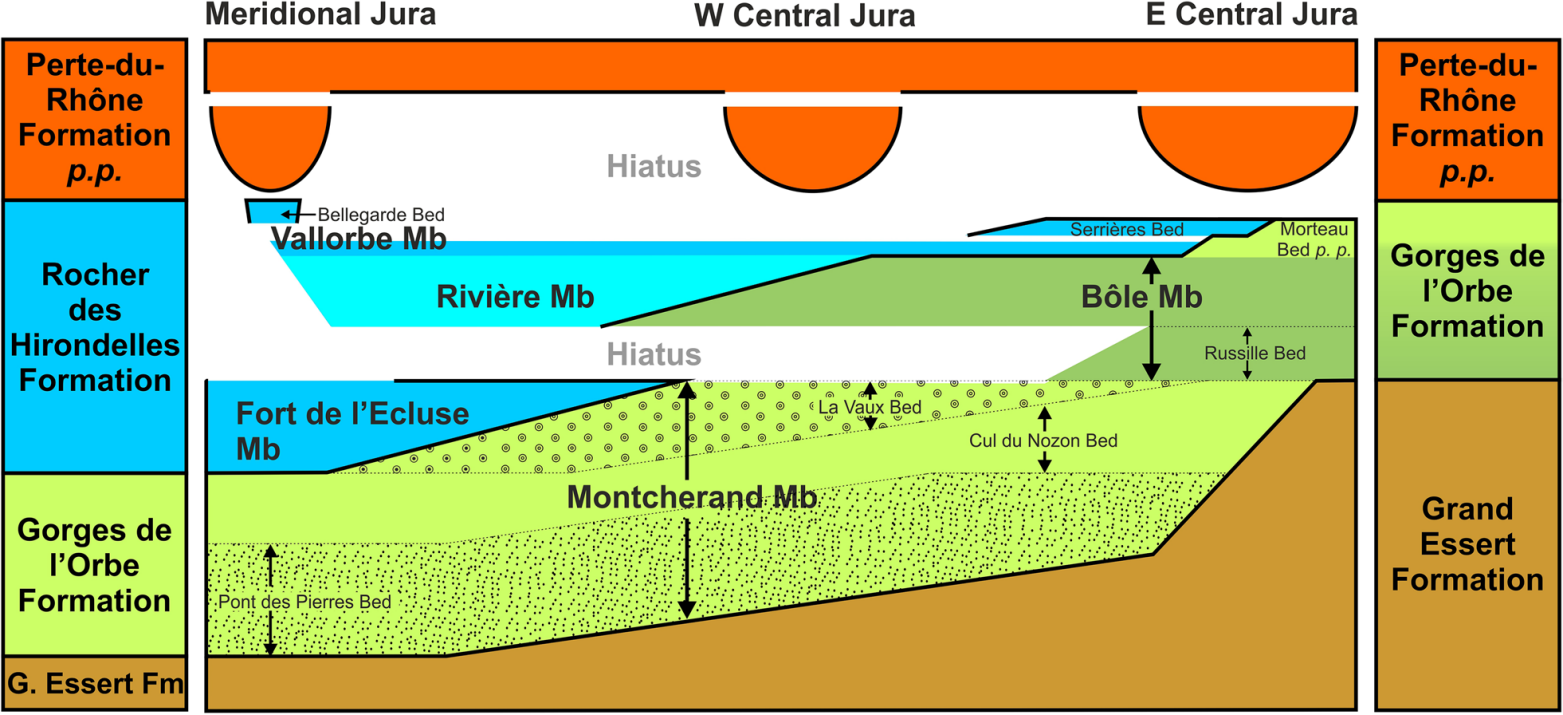
Synthetic scheme of the Hauterivian to Aptian lithostratigraphic units of the Jura Mountains following a SW to NE axis. This scheme shows well the strong diachronism of the presently studied formations and members and the lateral relationships between them

The sediments framed below by the Grand Essert Formation and above by the Perte-du-Rhône Formation are historically known as “Urgonien jaune”, “Urgonien inférieur” or Marnes de la Russille, and “Urgonian blanc”. These lithostratigraphic units were renamed in accordance with the rules of the International Stratigraphic Commission (ISC) (Fig. 26), in which each lithostratigraphic unit is defined by a geographic component, its lithological properties, and its stratigraphic relation (Hedberg, 1976; Remane et al., 2005).

Two new formation names were proposed in Strasser et al. (2016) in the framework of the HARMOS project to replace these three units. The lower one, the GDO Fm was established for outer platform environments (ex- Urgonien jaune sensu Remane, 1989) and the Vallorbe Formation for inner platform environments (Urgonien blanc or Urgonien supérieur). However due to the historical confusion concerning the position of the Marnes de la Russille, the correct interpretation of the “Urgonien inférieur” remained unclear. New field observations show that the “Urgonien inférieur” unit should be integrated to the GDO Fm as an upper member and that the “Marnes de la Russille” sensu Conrad and Masse (1989) should be incorporated to this unit rather than to the “Vallorbe Formation” in order to reconcile the divergent conceptions. Furthermore, the “Vallorbe Formation”, introduced in replacement of the “Urgonien blanc” unit, was defined on the type-section of the Vallorbe station which is not representative of the whole “Urgonien blanc” facies. For this reason, a new formation was designated at the Rocher des Hirondelles type section, in the Meridional Jura.

The GDO Fm comprises two members with the bottom marl and limestone of the Montcherand Member, and the top coral-echinoid-rich marly Bôle Mb. The RDH Fm comprises three members, from the base to the top: the first rudist-rich limestone unit of the Fort de l’Écluse Member; the *Heteraster*-rich marl and limestone of the Rivière Member; the second rudist-rich limestone unit of the Vallorbe Member.

A new sedimentological model based on numerous sections and fossils point to strong diachronic ages of lithostratigraphic units with a general progradation of a carbonate platform/ramp from a shallow, peritidal platform developed in the present-day Meridional Jura area toward external deeper-water shelf environments of the present-day Central Jura area and Molasse basin. This sedimentological model allows to put in relative agreement these two opposite schools with a late Hauterivian to early Barremian age of the Urgonien blanc facies in the Geneva region and a late Barremian age in the Central Jura.

## Abbreviations

MHNG: Muséum d’Histoire naturelle de Genève
GEPI: Département de géologie et de paléontologie
MGL: Musée de Géologie de Lausanne
